# Resident and Migratory Falcons' Breeding Phenology and Productivity Respond Differently to Weather and Climate Change Across the Arctic

**DOI:** 10.1111/gcb.71022

**Published:** 2026-08-05

**Authors:** Nick A. Gulotta, Knud Falk, Skip Ambrose, Nicholas W. Bakner, Peter J. Bente, Travis L. Booms, Kurt K. Burnham, Suzanne Carrière, Michael T. Henderson, Sergey Kharitonov, Alexander V. Kondratyev, Olga Kulikova, Peter Lindberg, Berth‐Ove Lindström, Svetlana Mechnikova, Dave Mossop, Søren Møller, Ólafur K. Nielsen, Kent Nilsson, Tuomo Ollila, Sture Orrhult, Ivan Pokrovsky, Marco Restani, Bryce W. Robinson, Robert N. Rosenfield, Ted Swem, Patrick H. Wightman, Nicholas P. Huffeldt

**Affiliations:** ^1^ Warnell School of Forestry and Natural Resources University of Georgia Athens Georgia USA; ^2^ Independent Researcher, vanderfalk.dk Stockholm Sweden; ^3^ Retired US fish and Wildlife Service Fairbanks Alaska USA; ^4^ University of Delaware Newark Delaware USA; ^5^ Alaska Dept. of Fish & Game Douglas Alaska USA; ^6^ High Arctic Institute Orion Illinois USA; ^7^ Environment and Natural Resources Government of the Northwest Territories Yellowknife Canada; ^8^ The Peregrine Fund The World Center for Birds of Prey Boise Idaho USA; ^9^ Bird Ringing Centre of Russia IEE RAS Moscow Russia; ^10^ Institute of Biological Problems of the North Feb RAS Magadan Russia; ^11^ Department of Biological and Environmental Sciences University of Gothenburg Göteborg Sweden; ^12^ Independent Researchers Boden Sweden; ^13^ Arctic Research Station of Institute of Plant and Animal Ecology Ural Branch Russian Academy of Sciences Labytnangi Russia; ^14^ Yukon Research Centre Whitehorse Yukon Canada; ^15^ Department of Science and Environment Roskilde University Roskilde Denmark; ^16^ Natural Science Institute of Iceland Garðabær Iceland; ^17^ Independent Researchers Dals Långed Sweden; ^18^ Peregrine Working Group Rovaniemi Finland; ^19^ Independent Researchers Ed Sweden; ^20^ Department of Migration Max Planck Institute of Animal Behavior Radolfzell Germany; ^21^ St. Cloud State University St. Cloud Minnesota USA; ^22^ The Peregrine Fund, and Cornell Lab of Ornithology Cornell University Ithaca New York USA; ^23^ University of Wisconsin Stevens Point Wisconsin USA; ^24^ U.S. Fish and Wildlife Service Soldotna Alaska USA; ^25^ Department of Birds and Mammals Greenland Institute of Natural Resources Nuuk Greenland; ^26^ Department of Biology Lund University Lund Sweden

**Keywords:** Arctic, climate change, *Falco peregrinus*, *Falco rusticolus*, phenology, productivity

## Abstract

Climate change is disproportionately warming the Arctic, leading to changes in weather patterns that affect breeding phenology for many Arctic species. Despite extensive research on non‐predatory birds, few studies have examined the impacts of climate change on higher trophic levels, especially those comparing breeding phenology and productivity between migratory and resident species. Our study used long‐term monitoring data from 11 sites monitoring migratory peregrine falcons ( *Falco peregrinus* ) and 9 sites monitoring resident gyrfalcons ( *Falco rusticolus* ) across the circumpolar Arctic. We used remotely sensed environmental data to explore how environmental conditions affect breeding phenology and productivity for both species. We analyzed 5546 peregrine and 2241 gyrfalcon hatch records and observed earlier hatch dates at 5 of 9 gyrfalcon sites and 4 of 11 peregrine sites. In the Low Arctic, peregrines advanced hatch dates by 0.41 days/decade and gyrfalcons by 1.62 days/decade, whereas in the Sub Arctic, we observed a trend toward later hatch dates for gyrfalcons. Greater spring temperatures and vegetation greenness consistently resulted in earlier hatch dates for both species. Earlier snowmelt was linked to earlier hatch dates for peregrines and higher productivity for both species. Our data suggest that long‐distance migrating peregrines advance their phenology less than resident gyrfalcons, aligning with other studies showing that long‐distance migrants possess more fixed annual schedules and are less likely to align with variations in spring green‐up compared to residents. Contrary to our hypothesis, greater summer temperatures reduced productivity in both species. Given our findings, we recommend more detailed research on how summer temperatures affect productivity, as nestlings could suffer higher mortality from heat prostration than previously anticipated. Our findings indicate that while climate change affects breeding timing and productivity, the impact varies, suggesting increased weather variability could lead to more unpredictable responses in falcon breeding ecology in the Arctic.

## Graphical abstract

Climate change is altering breeding phenology and productivity of peregrine falcons and gyrfalcons across the circumpolar Arctic. Long‐term monitoring shows that responses vary among study areas and Arctic regions, with some populations advancing hatch dates while others show weaker or contrasting trends, highlighting spatially variable effects of environmental change.

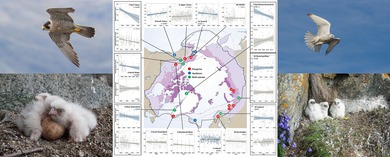

## 1 Introduction

With the rapid increase in global temperatures, the Arctic has warmed up to four times faster than the global rate over the past five decades (Masson‐Delmotte et al. 2021; Rantanen et al. 2022) and predictions from climate models indicate that temperatures in the Arctic will continue rising approximately 2.5 times faster than the global average (Masson‐Delmotte et al. 2021). Widespread Arctic warming has led to marked shifts in the timing of key seasonal events, likely contributing to accelerated phenological changes (Badeck et al. 2004; Post et al. 2018), including notable advances in the spring phenology of birds, arthropods, and plants (Høye et al. 2007; Dickey et al. 2008; Tulp and Schekkerman 2008). The changes in phenology are primarily driven by a bottom‐up mechanism, where increased temperatures result in an earlier snow melt, leading to earlier plant growth and emergence of arthropods (Stone et al. 2002; Tulp and Schekkerman 2008; Assmann et al. 2019). Prior work has demonstrated earlier springs and longer growing seasons have expanded the breeding window in the short Arctic summer for numerous avian species (Walther et al. 2002; Dickey et al. 2008; Lameris et al. 2018). Despite the growing evidence that environmental change disrupts the timing of reproduction in non‐predatory Arctic species that depend on synchrony with peak food availability (Saalfeld and Lanctot 2017; Saalfeld et al. 2019), the responses of top avian predators to Arctic warming remain largely unknown. Given that predatory birds depend on many of the same ecological cues as non‐predatory species, they may similarly be expected to adjust their phenology in response to shifting environmental conditions.

Migratory and resident birds attempt to time the hatching of their nestlings to coincide with periods of abundant food to enhance their offspring's chances of survival (Lack 1968). Despite this, the timing of breeding in many migratory birds shows limited flexibility, being more rigidly controlled by responses to external environmental cues, such as photoperiod, or an endogenous circannual rhythm aligned to the annual cycle (Dawson et al. 2001; Gwinner 2003, 2012; Åkesson and Helm 2020). In line with these expectations, resident and short‐distance migrants tend to cue their annual schedules directly to local environmental conditions and show greater adjustments of their breeding phenology to climate warming and advancing spring green‐up, whereas long‐distance migrants follow more fixed, internally driven schedules and are less able to align laying and hatching with changing environmental conditions on their breeding grounds (Huffeldt 2021; Kluen et al. 2017; Usui et al. 2017; Samplonius et al. 2018; Søraker et al. 2022). As a result, several long‐distance migratory species now exhibit phenological mismatches between the timing of hatching and the period of maximum food availability, which has been shown to negatively influence demography (Dickey et al. 2008; Clausen and Clausen 2013; Doiron et al. 2015; Kwon et al. 2019; Saalfeld et al. 2019). Nonetheless, research on the influence of climate change on the reproductive phenology of Arctic birds has largely focused on a narrow set of primarily herbivorous or insectivorous migrants, with relatively few studies on predatory bird species and even fewer comparing species with contrasting life‐history strategies (e.g., resident vs. migrants). Consequently, we still know little about how climate change shapes the breeding phenology of avian top predators, or how climate sensitivity compares between resident and migratory predatory bird species (Dickey et al. 2008; Clausen and Clausen 2013; Doiron et al. 2015; Kwon et al. 2019; Saalfeld et al. 2019).

Here our research aims to bridge these gaps by examining the effects of climate change on the breeding phenology and productivity of two avian top predators with circumpolar distributions and contrasting life‐history strategies: the migratory peregrine falcon ( *Falco peregrinus* , hereafter “peregrine”) and resident gyrfalcon ( *Falco rusticolus* ). In the Arctic, both species face harsh conditions before laying eggs and, in some regions, throughout the entire nesting stage. Gyrfalcons, which remain in Arctic or Boreal regions year‐round, often begin nesting amidst substantial snow cover and temperatures below the freezing point for long periods (Platt 1976; Poole and Bromley 1988; Booms et al. 2020; Henderson et al. 2021). Across their shared breeding range from the Sub Arctic to the High Arctic, peregrines and gyrfalcons have protracted breeding cycles, with nesting stages lasting approximately 120 and 160 days, respectively (Booms et al. 2020; White et al. 2024). Peregrines migrating from southern latitudes track the northward retreat of snow to reach their Arctic breeding areas and frequently encounter snowstorms en route (Curk et al. 2020), with High Arctic breeders traveling 200–300 km per day before arrival at the breeding grounds (Burnham et al. 2012). The constraints of a long nesting cycle, combined with a narrow window for successful reproduction and strong latitudinal variation in spring conditions, may make it particularly difficult for long‐distance migratory peregrines to adjust their breeding phenology (Visser et al. 2004). In contrast, resident gyrfalcons, which remain on or near their Arctic breeding grounds year‐round, are likely better suited to fine‐tune their breeding phenology to local environmental change, mirroring patterns reported for other resident bird species (Kluen et al. 2017; Samplonius et al. 2018; Søraker et al. 2022). Nonetheless, both species are exposed to climate‐driven shifts in local conditions that can directly influence breeding phenology and productivity; yet, despite evidence of such changes in some populations (Burnham and Burnham 2011; Carrière and Matthews 2013; Anctil et al. 2014; Carlzon et al. 2018; Swem and Matz 2018), there has been no comprehensive assessment of how local environmental variation shapes the timing of breeding and productivity of gyrfalcons and peregrines across the circumpolar region.

Our objectives for this study were to: (1) investigate variation in breeding phenology across Arctic regions for both species; (2) determine factors affecting the breeding phenology, specifically analyzing the effects of spring temperature, snow cover, and vegetation greenness (as indicated by the normalized difference vegetation index, NDVI) on breeding phenology at both the individual monitoring site level and across the Arctic regions; (3) explore changes in falcon productivity over time, focusing on the influence of hatching dates, summer temperatures, snow cover, NDVI, and precipitation at the Arctic regional level. We predicted that snow cover would influence hatch dates more significantly for peregrines than for gyrfalcons given gyrfalcons' tendency to initiate breeding while winter conditions still prevail. We also predicted that warmer spring temperatures and greater NDVI would be associated with earlier hatch dates in both species, with resident gyrfalcons showing the stronger phenological response. Gyrfalcons are likely more tightly coupled to local conditions and rely on resident ptarmigan (*Lagopus* spp.) as prey (Nielsen 2003; Booms et al. 2020). As a result, gyrfalcons may be more responsive to warming than migratory peregrines, which arrive later and depend mainly on migratory passerine and waterbird prey (Falk et al. 1986; Court et al. 1988; White et al. 2024). Regarding annual falcon productivity, we predicted that increased precipitation and lower temperatures would reduce productivity, as heavy rainfall has been linked to nestling mortality through rapid heat loss (Poole and Bromley 1988; Bradley et al. 1997; Anctil et al. 2014; Carlzon et al. 2018; Booms et al. 2020). Consistent with previous studies on other bird species (Perrins 1970; Verhulst and Nilsson 2008), we expected earlier hatch dates to be associated with higher productivity, and likewise predicted that earlier snowmelt would facilitate more efficient prey capture and increase productivity in both species.

## 2 Methods

### 2.1 Study Sites

We followed the Conservation of Arctic Flora and Fauna (CAFF) definition of Arctic (Christensen et al. 2013) and (Hohn and Jaakkola 2010) definition of Sub, Low, and High Arctic. Monitoring of falcon populations was carried out at 16 peregrine study sites and at 9 gyrfalcon sites (Figure 2, Table S1). The study sites were distributed across the Arctic regions as follows: 5 peregrine study sites were located in the Sub Arctic, 8 in the Low Arctic, and 3 in the High Arctic. For gyrfalcons, 4 study sites were located in the Sub Arctic, 4 in the Low Arctic, and 1 site in the High Arctic (see Figure 2 for distribution of study sites across the Arctic regions and nations and Table S1 for site characteristics). Study sites that provided data for short time spans were only included in determining timing of breeding in the different Arctic zones (total *n* = 16); for trend analyses we selected the long‐term data sets, which we consider as ≥ 9 monitoring years, which were available for 11 peregrine falcon study sites and all 9 gyrfalcon sites. We refer to ‘sites’ for monitoring/study sites—which encompass many individual falcon territories.

### 2.2 Estimating Hatch Dates and Productivity

Hatch dates were estimated from data collected during visits to falcon nests for investigating productivity (Franke et al. 2020) when the age of each of the nestlings was also recorded. Ageing of the nestlings was based on published aging guides, which use morphometric measurements (i.e., wing length and/or tail measurement), photo guides and descriptions of known nestling ages, and nest camera traps (Poole 1989; Ratcliffe 1993; Clum et al. 1996; Anderson et al. 2017). The hatch date, expressed as day of the year (DoY), was then estimated by back‐calculating from the age of the young; in all analyses we use the age of the first‐hatched nestling in each brood. Annual productivity was measured as number of large/fledged young per occupied territory recorded at each monitoring site (Anderson et al. 2017), reusing the dataset analyzed by Franke et al. (2020) and selecting the data for the study sites where also phenology data were available.

### 2.3 Environmental Covariates

There are a limited number of climate stations in the Arctic that offer continuous observations over extended durations. We therefore used remote sensing data extracted from Google Earth Engine to address our questions related to the influence of environmental covariates on breeding phenology and productivity (Gorelick et al. 2017). To ensure environmental covariates were biologically relevant, we defined all temporal windows relative to species‐specific breeding stages (egg‐laying/incubation, hatching/brood rearing), which were estimated from site‐specific hatch date distributions.

To pinpoint the different time windows relevant for selecting environmental data to include in the analyses, we initially gathered summary statistics of nestling hatch dates from each study site, and calculated the 1st quantile, median, and 3rd quantile of nestling hatch dates (DoY). From these quantiles, we subtracted the known average incubation durations—33 days for peregrine falcon (White et al. 2024) and 36 days for gyrfalcon (Booms et al. 2020)—to estimate the respective start of incubation, i.e., the approximate egg‐laying stage. To estimate the impact of temperature during the egg‐laying stage (spring temperature), we then used mean monthly temperatures that overlapped with the back‐calculated egg‐laying stage, and if back‐calculated quantiles fell across two different months, we took the mean across both months for analysis. We obtained temperature data from the ERA5‐land monthly reanalysis data, which has a resolution of 0.1° × 0.1° (Hersbach et al. 2020). For each study site, we constructed a 50 km circular buffer around a centralized coordinate provided by each data contributor, roughly covering the size of each monitoring site (Table S1), from which we extracted temperature data. Moreover, to assess the impact of temperature on productivity, we employed a similar methodology as stated above, extracting temperature (summer temperature) data based on hatch date quantiles.

Subsequently, we explored the impact of snow cover on nestling hatch dates and productivity for both peregrines and gyrfalcons, focusing on the timing of snowmelt as a key constraint on breeding initiation. To investigate these effects, we applied a method similar to our temperature extraction; within the 50 km circular buffer areas we extracted snow cover data from the ERA5‐daily reanalysis dataset with a resolution of 0.1° × 0.1° (Hersbach et al. 2020). For our purposes, we established the onset of minimal snow cover as the first day of year during a three‐week period on which snow cover was reduced to below 20% within the designated buffer areas, representing a sustained transition to snow‐free conditions relevant for breeding and minimizing the influence of short‐term fluctuations in snow cover (Fauchald et al. 2017).

We then attempted to determine whether nestling hatch dates were advancing with increased spring vegetation greenness, as measured by NDVI, a phenomenon reported in numerous Arctic‐breeding species (Van Der Jeugd et al. 2009; Ross et al. 2017, 2018; Lameris et al. 2018). Additionally, we sought to understand whether higher NDVI values at the time of respective hatch dates could predict greater productivity. To address these questions, we accessed the Global Inventory Modeling and Mapping Studies (GIMMS) NDVI dataset, sourced from the Advanced Very High‐Resolution Radiometer (AVHRR) sensors aboard National Oceanic and Atmospheric Administration (NOAA) satellites. Following the approach described previously, we extracted NDVI values for the hatch period at each study site to evaluate whether hatch dates advanced with increasing vegetation greenness and whether vegetation conditions during the hatch period predicted productivity. Because the hatch window occasionally spanned two calendar months, we averaged NDVI values across both months for analysis, following the same approach used for temperature data.

Lastly, since several studies have demonstrated that precipitation during the brood rearing stage negatively impacts reproductive success of Arctic breeding peregrines (Bradley et al. 1997; Anctil et al. 2014; Carlzon et al. 2018) and gyrfalcons (Poole and Bromley 1988; Booms et al. 2020), we asked whether this phenomenon was occurring across their circumpolar distribution. To do this, we extracted precipitation data at the study site level for months when nestlings hatched, employing the same approach as above using the ERA5‐monthly reanalysis dataset.

### 2.4 Statistical Analysis

We conducted our analysis using R version 4.3.1 (R Development Core Team 2024), fitting all models described below using the brms package with a Gaussian error distribution and the default priors (Bürkner 2017). Each analysis is numbered below and corresponds directly to model descriptions in Table S2 and associated figures.

Prior to modelling, we centered and scaled all numerical fixed effects to ease interpretation and aid model convergence. We used untransformed response variables in all models, as each was a continuous measure that followed a normal distribution. For all models, we did not use model selection techniques to rank candidate models because the relative effect of all covariates was of interest. Instead, we checked for multicollinearity using the package Performance (Lüdecke et al. 2021), and for all models the parameter estimates and associated variance inflation factors were < 10 (O'brien 2007; James et al. 2013). Moreover, for all analyses involving environmental covariates, we selected hatch dates from 1981 forward to ensure completeness in covariate data, given that NDVI data was unavailable before 1981. For all linear mixed effect models, we performed 10,300 iterations of each model with a burn in of 300, a thinning interval of 10, and we used two Markov chains per model. Model convergence was evaluated through visual inspection of trace and density plots, along with R‐hat statistics, where values less than 1.1 were taken as evidence of adequate convergence. For each model, we present the posterior median (*β*), 95% credible intervals (CrI), and the probability of direction (PD), which reflects the proportion of the posterior that aligns with the estimated effect direction (Makowski, Ben‐Shachar, Chen, and Lüdecke 2019; Makowski, Ben‐Shachar, and Lüdecke 2019). We interpreted effects with 95% credible intervals (CrI) not overlapping zero as the strongest evidence, and used the probability of direction (PD) as a continuous measure of directional support to assess the existence of an effect, particularly when credible intervals overlapped zero (Makowski, Ben‐Shachar, Chen, and Lüdecke 2019; Makowski, Ben‐Shachar, and Lüdecke 2019). Higher PD values indicate greater consistency in effect direction; for example, PD values around 89% reflect that approximately 89% of the posterior distribution aligns with the estimated effect direction. We describe such cases as providing moderate directional support, while avoiding strict threshold‐based classification (Weller et al. 2022; Johansson et al. 2024).

#### 2.4.1 Analysis 1: Regional Differences in Breeding Phenology

To quantify differences in breeding phenology between peregrines and gyrfalcons breeding in different Arctic regions. We constructed a single Bayesian linear mixed effect model using untransformed values of hatch dates (DoY) as the response variable, including fixed effects of Arctic region (Sub Arctic, Low Arctic, High Arctic), species (peregrine, gyrfalcon), and an interaction between Arctic region and species. To account for variability between study sites and annual fluctuations, we incorporated a random effect for study site and year in the analysis (see Table S3 for model outputs).

#### 2.4.2 Analysis 2: Effects of Year and Environmental Covariates on Breeding Phenology at Study Sites

We then evaluated the influence of year and environmental factors on breeding phenology separately at each long‐term study site (those with at least 9 years of data). We used Bayesian linear mixed effects models for each study site and respective species (11 peregrine study sites, 9 gyrfalcon study sites; see Table S2), and fitted untransformed values of hatch dates (DoY) as the response variable and fitted fixed effects of year, spring temperature during the egg‐laying stage, first day of minimal snow cover (snow cover < 20%), and NDVI during the hatching stage. To account for the variance across breeding territories, we fitted a random intercept for territory.

#### 2.4.3 Analysis 3: Effects of Year and Environmental Covariates on Breeding Phenology Across Arctic Regions

The framework described in “Analysis 2” was then extended to explore how environmental conditions affect breeding phenology across different Arctic regions (Sub Arctic, Low Arctic) for each species, and we combined data from each Arctic region for each species to generate pooled estimates (see Table S2). However, we lacked adequate data to evaluate the effects on both species in the High Arctic, as there were too few study sites in this region. To capture variation among study sites and among territories within study sites, we adjusted our random effect structure and modeled territory (Territory ID) as nested within study sites (Monitoring site ID) using nested random intercepts.

#### 2.4.4 Analysis 4: Effects of Year and Environmental Covariates on Productivity Across Arctic Regions

Finally, we assessed how environmental conditions affect the productivity of both peregrines and gyrfalcons. Employing a method akin to the one previously described for analyzing the influence of environmental conditions for each Arctic region (see “Analysis 3”), we aggregated data for each species from study sites classified as either Sub Arctic or Low Arctic. Due to the lack of productivity data from High Arctic study sites, we were unable to evaluate the impact of environmental conditions on productivity in these areas. Nevertheless, by combining data from all Arctic regions, we were able to derive an overall productivity estimate for each species. To do this, we fitted untransformed values of productivity as the response variable (productivity was a continuous measure) and fitted fixed effects of year, average yearly hatch date for each respective study site, snow cover, summer temperature, summer precipitation, and NDVI. Note that productivity data may be biased slightly upwards due to many surveys mainly covering the nestling stage (Franke et al. 2020).

## 3 Results

### 3.1 Regional Differences in Breeding Phenology

**FIGURE 1:**
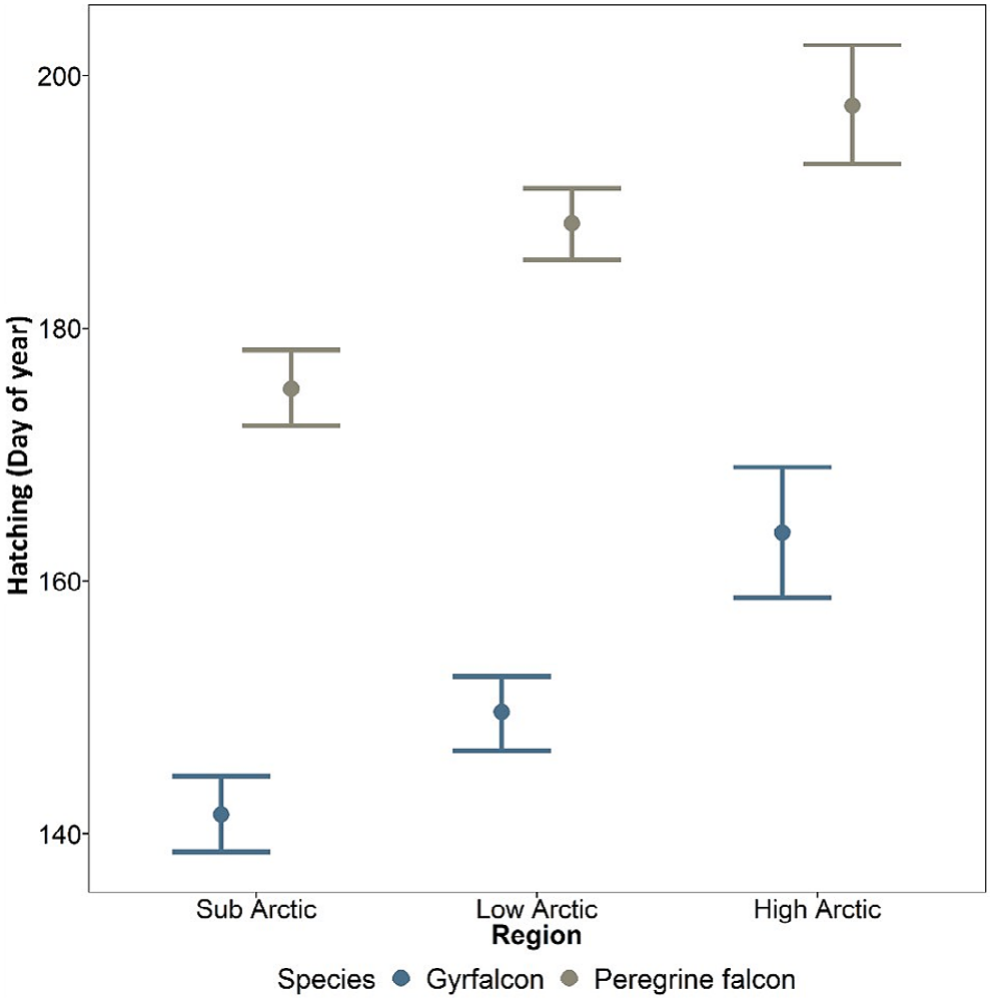
Univariate results for sources of variation in hatch dates among Arctic regions for peregrines and gyrfalcons (Analysis 1; see Section 2). The plot depicts an interaction between the Arctic region (Sub, Low, High Arctic) and species (peregrines, gyrfalcon). The study period covers 1967–2022 for peregrines and 1973–2022 for gyrfalcons. Mean hatch date (day 140 = 20 May; day 200 = 19 July) of the first egg in nests of peregrines and gyrfalcons in different zones of the Arctic.

We had a total of 5546 hatch dates for peregrines (*n* = 3787 Sub Arctic, *n* = 1674 Low Arctic, *n* = 85 High Arctic) and a total of 2241 hatch dates for gyrfalcons (*n* = 1310 Sub Arctic, *n* = 863 Low Arctic, *n* = 68 High Arctic). Peregrines generally hatched 34 days later than gyrfalcons and this pattern held true across each Arctic region (Table S3; Figure 1); when comparing hatch dates on the six study sites where both species were surveyed, gyrfalcons hatched from 21 to 43 days earlier than peregrines (Table 1). For both species, hatch dates were earliest in the Sub Arctic, intermediate in the Low Arctic, and the latest hatch dates were observed in the High Arctic (Table S3; Figure 1).

**TABLE 1:** Univariate results for the effect of environmental covariates on hatch dates among all long‐term study sites for peregrines (*n* = 11) and gyrfalcons (*n* = 9) in the period 1981–2022 (Analysis 2; see Section 2).

Study site	Site number	Intercept	Year		Snow		Temperature		NDVI	
		*β* (95% CrI)	*β* (95% CrI)	PD (%)	*β* (95% CrI)	PD (%)	*β* (95% CrI)	PD (%)	*β* (95% CrI)	PD (%)
Peregrine										
South Greenland	2	185.59 (184.31, 187.03)	−0.25 (−1.02, 0.50)	73.77	0.48 (−0.71, 1.70)	78.62	−0.70 (−1.99, 0.55)	86.16	**−0.55 (−1.29, 0.21)**	92.08
Central West Greenland	3	190.47 (189.84, 191.12)	−0.84 (−1.38, −0.29)*	99.82	**0.50 (−0.22, 1.21)**	91.62	0.30 (−1.07, 0.48)	77.72	0.00 (−0.52, 0.53)	50.31
North Greenland	4	197.89 (195.18, 200.44)	1.85 (−1.11, 4.81)	88.73	−0.60 (−2.42, 1.19)	75.43	−0.02 (−1.65, 1.60)	51.12	0.62 (−2.35, 3.65)	66.67
Mackenzie River (Ca)	5	180.29 (179.61, 180.97)	**−0.98 (−2.27, 0.31)**	92.86	2.60 (0.93, 4.28)*	99.97	1.74 (0.50, 2.94)*	99.68	**0.59 (−0.11, 1.30)**	95.09
North Slope Yukon (Ca)	6	181.55 (179.38, 183.71)	0.17 (−2.44, 2.69)	54.96	−7.17 (−11.59, −2.61)*	99.83	−8.74 (−14.15, −2.98)*	99.89	−0.61 (−3.80, 2.65)	64.84
Yukon/Peel Rivers (Ca)	7	171.33 (170.54, 172.14)	−2.99 (−3.97, −2.03)*	100	0.78 (−0.59, 2.17)	86.30	0.35 (−1.11, 1.81)	67.54	−0.91 (−1.76, −0.05)*	98.24
Upper Yukon (US)	9	170.13 (169.58, 170.69)	0.62 (0.18, 1.06)*	99.69	0.68 (0.27, 1.10)*	99.90	−0.50 (−0.95, −0.04)*	98.51	0.07 (−0.31, 0.45)	64.33
Colville River (US)	10	187.47 (186.78, 188.18)	**−0.29 (−0.74, 0.17)**	89.20	2.12 (1.35, 2.89)*	100	0.35 (−0.41, 1.12)	80.94	0.46 (0.01, 0.92)*	97.74
Schuchya River (Ru)	14	188.60 (187.35, 189.86)	0.05 (−1.12, 1.17)	53.16	2.51 (0.46, 4.61)*	99.15	0.18 (−1.66, 2.05)	57.21	−0.25 (−1.50, 1.01)	65.39
Lapland (Fi)	18	175.64 (175.05, 176.21)	0.59 (0.31, 0.86)*	100	1.00 (0.71, 1.28)*	100	−1.13 (−1.47, −0.78)*	100	0.00 (−0.29, 0.29)	50.08
Norrbotten (Se)	19	168.86 (168.06, 169.68)	−0.19 (−0.67, 0.30)	78.03	0.88 (0.21, 1.55)*	99.45	0.32 (−0.36, 0.99)	82.16	−0.53 (−1.03, −0.03)*	98.17
Gyrfalcon										
Iceland	1	147.95 (146.99, 148.93)	0.11 (−0.70, 0.89)	60.41	0.93 (0.20, 1.65)*	99.49	**−0.74 (−1.55, 0.05)**	96.63	−0.93 (−1.74, −0.11)*	98.61
Central West Greenland	3	148.90 (145.09, 152.72)	**−7.21 (−16.21, 1.63)**	94.35	**−4.33 (−9.87, 1.19)**	93.95	0.74 (−3.06, 4.50)	65.15	−2.14 (−6.45, 2.24)	82.91
North Greenland	4	163.70 (161.40, 165.92)	−3.61 (−5.92, −1.27)*	99.81	**1.25 (−0.52, 3.03)**	92.04	−1.54 (−3.13, 0.02)*	97.37	**−1.89 (−4.33, 0.47)**	94.38
North Slope Yukon (Ca)	6	150.08 (149.07, 152.58)	−2.62 (−4.53, −0.75)*	99.71	6.25 (3.09, 9.43)*	100	−0.55 (−2.01, 0.88)	76.94	4.95 (1.74, 8.10)*	99.83
Peel Yukon Rivers (Ca)	7	155.50 (150.56, 160.27)	−14.96 (−22.67, −7.78)*	100	**−3.47 (−8.00, 1.00)**	93.81	0.66 (−3.80, 4.80)	62.38	−5.97 (−9.41, −2.43)*	99.90
South Yukon (Ca)	8	141.58 (139.69, 143.64)	2.65 (1.06, 4.28)*	99.91	−0.94 (−2.42, 0.62)	88.50	−2.89 (−4.46, −1.27)*	100	**−1.23 (−2.66, 0.16)**	95.81
Seward Peninsula (US)	11	153.21 (152.32, 154.17)	**−1.21 (−2.62, 0.21)**	95.19	−0.66 (−2.02, 0.68)	82.65	−3.66 (−5.68, −1.63)*	99.99	0.22 (−1.63, 2.11)	59.46
Schuchya River (Ru)	14	148.72 (145.96, 151.79)	0.48 (−2.05, 2.96)	64.74	−1.34 (−4.85, 2.13)	78.50	**−1.65 (−4.08, 0.79)**	91.12	−3.78 (−7.30, −0.23)*	98.17
Lapland (Fi)	18	137.18 (135.55, 138.96)	−0.27 (−1.93, 1.37)	62.28	−0.48 (−2.77, 1.81)	65.95	−1.37 (−4.11, 1.36)	83.74	0.13 (−1.65, 1.90)	55.65

*Note:* We report beta estimates (*β*) as posterior medians, 95% credible intervals (CrI), and probability of direction (PD). Negative *β* values indicate that the covariate influences towards earlier hatch dates, and positive values later hatch dates; *β* values for intercept can be read as modelled ‘day of year’ of average hatch date. Site numbers correspond to maps presented in Figures 2, 3, 4, 5. Effects where credible intervals did not overlap zero were interpreted as having the strongest directional support. Effects where credible intervals overlapped zero but PD was ≥ 89% were interpreted as having moderate directional support. Both are highlighted in bold, with effects having the strongest directional support additionally marked with an asterisk. All covariates were standardized prior to analysis.

### 3.2 Effects of Year and Environmental Covariates on Breeding Phenology

**FIGURE 2:**
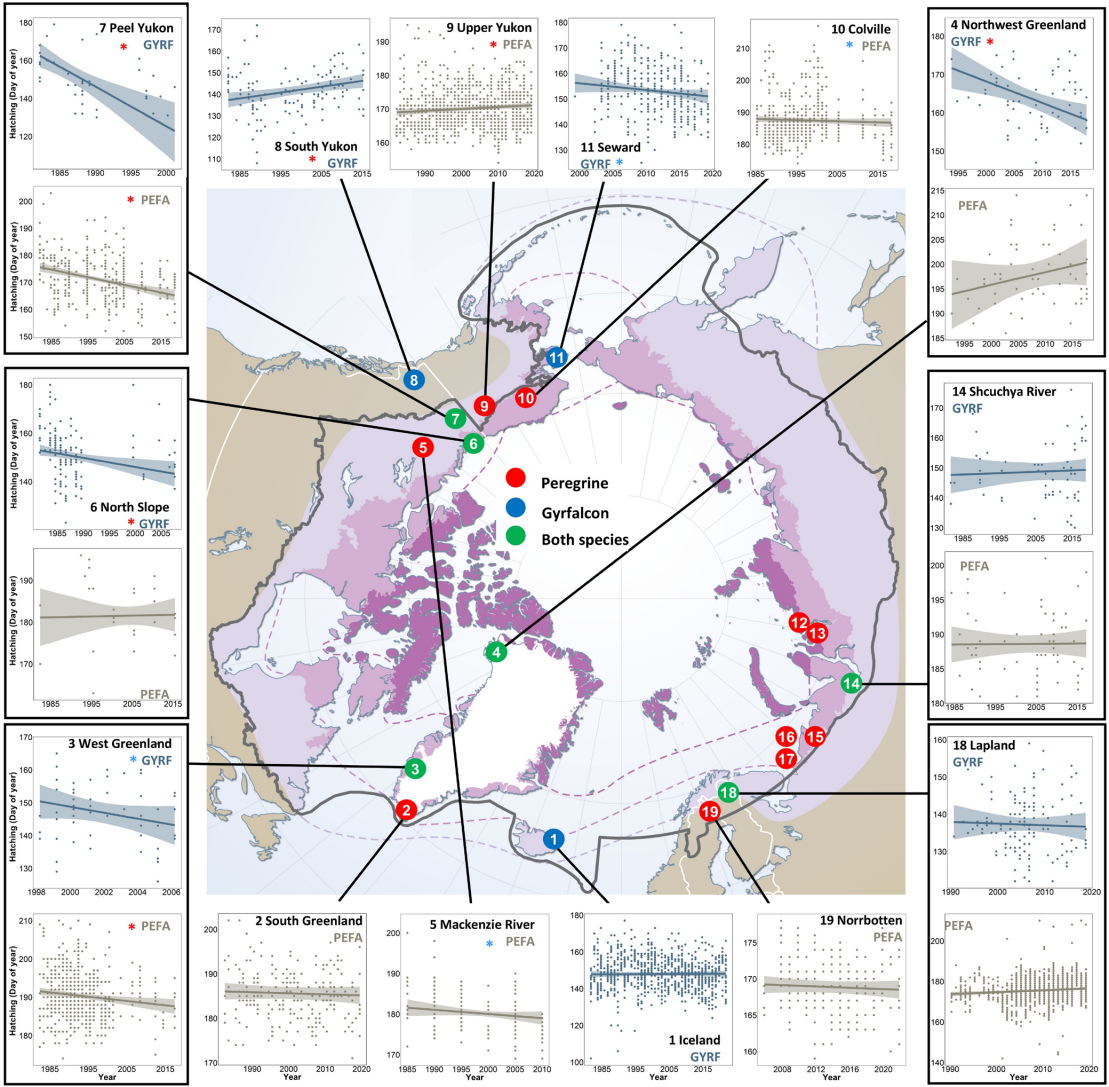
The location of all study sites, and univariate results for the effect of year on hatch dates (individual nest records) across each respective long‐term monitoring site (≥ 9 years) for peregrines (PEFA) and gyrfalcons (GYRF) (Analysis 2; see Section 2). The overall study period covers 1981–2022; note different axis units. Light purple refers to Sub Arctic, medium purple to Low Arctic, and dark purple to High Arctic as defined by Hohn and Jaakkola (2010) and the solid line is the boundary for Conservation of Arctic Flora and Fauna (CAFF) biodiversity monitoring plans (Christensen et al. 2013). Red stars indicate effects where 95% credible intervals did not overlap zero, reflecting the strongest evidence for an effect, while blue stars indicate effects with moderate directional support where credible intervals overlapped zero but probability of direction values suggested consistent directional trends.

**FIGURE 3:**
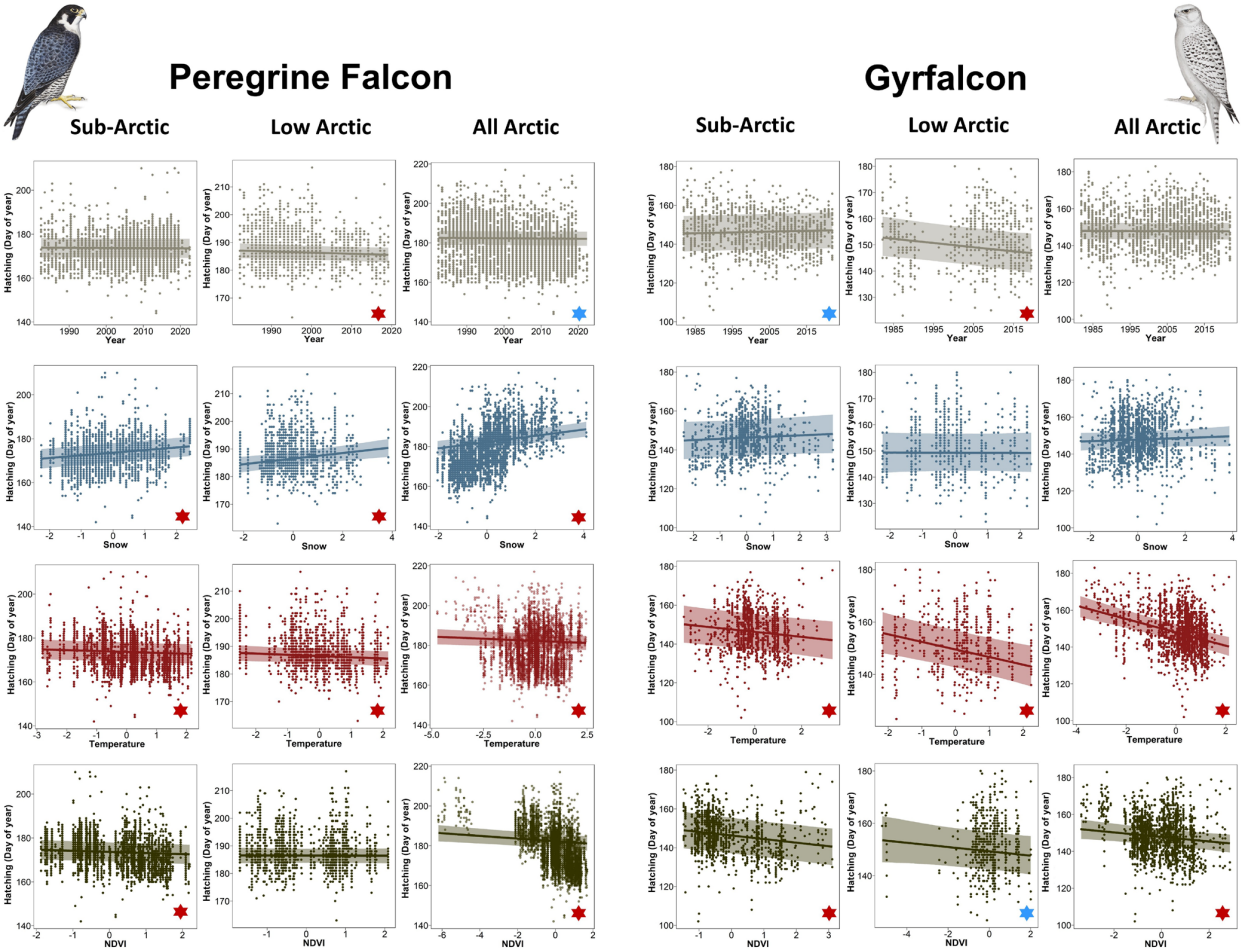
Univariate results for the effect of year and environmental covariates on hatch dates among Arctic regions for peregrines and gyrfalcons (Analysis 3; see Section 2). The study period covers 1981–2022 for both peregrines and gyrfalcons. Red stars indicate effects where 95% credible intervals did not overlap zero, reflecting the strongest evidence for an effect, while blue stars indicate effects with moderate directional support where credible intervals overlapped zero but probability of direction values suggested consistent directional trends.

For both species, we detected long‐term changes in breeding phenology, but with variable trends across the circumpolar Arctic (Figure 2; Table 1). Across peregrine study sites, two of four study sites showed strong support for earlier hatch dates over time (CrI not overlapping zero), while two study sites showed moderate directional support, with most of the posterior distribution aligned with earlier hatch dates (Table 1; Figure 2). Similarly, among gyrfalcon sites, three showed strong support, and two additional sites showed moderate directional support with the majority of posterior mass indicating earlier hatch dates (Table 1; Figure 2). Overall, four of 11 peregrine sites and five of nine gyrfalcon sites demonstrated a negative year effect, indicating earlier hatch dates over time (Table 1; Figure 2). Conversely, we observed a delayed hatch date trend over time at two of the 11 study sites for peregrines and one of the nine sites for gyrfalcons, as indicated by a positive year effect (Table 1; Figure 2). When data were consolidated at the Arctic regional level, we found a consistent negative year effect in the Low Arctic for both peregrines (*β* = −0.39, 95% CrI = −0.74, −0.01, PD = 98.54%) and gyrfalcons (*β* = −2.15, 95% CrI = −3.91, −0.23, PD = 98.62%); this translates into ~0.41 days/decade earlier hatch dates for peregrines and ~1.62 days/decade earlier for gyrfalcons. Conversely, in the Sub Arctic, a positive year effect was observed for gyrfalcons (*β* = 0.49, 95% CrI = −0.20, 1.18, PD = 91.78%), with the posterior distribution largely aligned with later hatch dates over time, suggesting a modest trend toward delayed hatching (0.42 days/decade later; Table 2; Figure 3). Pooled data from all Arctic regions suggested a slight shift toward earlier hatch dates in peregrines, with the posterior distribution largely aligned with earlier timing (0.13 days/decade earlier; Figure 3).

**TABLE 2:** Univariate results for the effect of year and environmental covariates on hatch dates among Arctic regions for Peregrines and Gyrfalcons in the period 1981–2022 (Analysis 3; see Section 2).

Species	Region	Intercept		Year		Snow		Temperature		NDVI	
		*β* (95% CrI)	PD (%)	*β* (95% CrI)	PD (%)	*β* (95% CrI)	PD (%)	*β* (95% CrI)	PD (%)	*β* (95% CrI)	PD (%)
Peregrine Falcon	Sub Arctic	173.65 (169.28, 178.00)	100	−0.08 (−0.34, 0.18)	72.03	1.20 (0.92, 1.47)*	100	−0.41 (−0.69, −0.14)*	99.87	−0.50 (−0.78, −0.22)*	99.97
	Low Arctic	186.51 (183.90, 188.99)	100	−0.39 (−0.74, −0.04)*	98.54	1.01 (0.46, 1.57)*	99.98	**−0.44 (−0.88, 0.00)**	97.45	−0.02 (−0.59, 0.55)	53.26
	Pooled	182.30 (178.59, 185.92)	100	**−0.13 (−0.34, 0.08)**	89.18	1.57 (1.24, 1.91)*	100	−0.43 (−0.69, −0.17)*	99.94	−0.66 (−1.03, −0.31)*	99.99
Gyrfalcon	Sub Arctic	146.23 (137.07, 155.37)	100	**0.49 (−0.20, 1.18)**	91.78	0.61 (−0.49, 1.72)	85.52	−1.29 (−2.22, −0.36)*	99.64	−1.86 (−2.80, −0.91)*	100
	Low Arctic	150.00 (143.17, 157.36)	100	**−2.15 (−3.91, −0.38)**	99.16	−0.02 (−1.20, 1.15)	51.24	−2.91 (−4.29, −1.53)*	100	**−0.82 (−1.91, 0.25)**	93.26
	Pooled	147.95 (143.49, 152.31)	100	−0.05 (−0.64, 0.52)	57.82	0.45 (−0.38, 1.30)	85.86	−3.62 (−4.73–2.52)*	100	−1.23 (−2.02, −0.45)*	99.91

*Note:* We report beta estimates (*β*) as posterior medians, 95% credible intervals (CrI), and probability of direction (PD). *β* values for intercept can be read as modelled ‘day of year’ of average hatch date. All covariates were standardized prior to analysis. Effects where credible intervals did not overlap zero were interpreted as having the strongest directional support. Effects where credible intervals overlapped zero but PD was ≥ 89% were interpreted as having moderate directional support. Both are highlighted in bold, with effects having the strongest directional support additionally marked with an asterisk.

**FIGURE 4:**
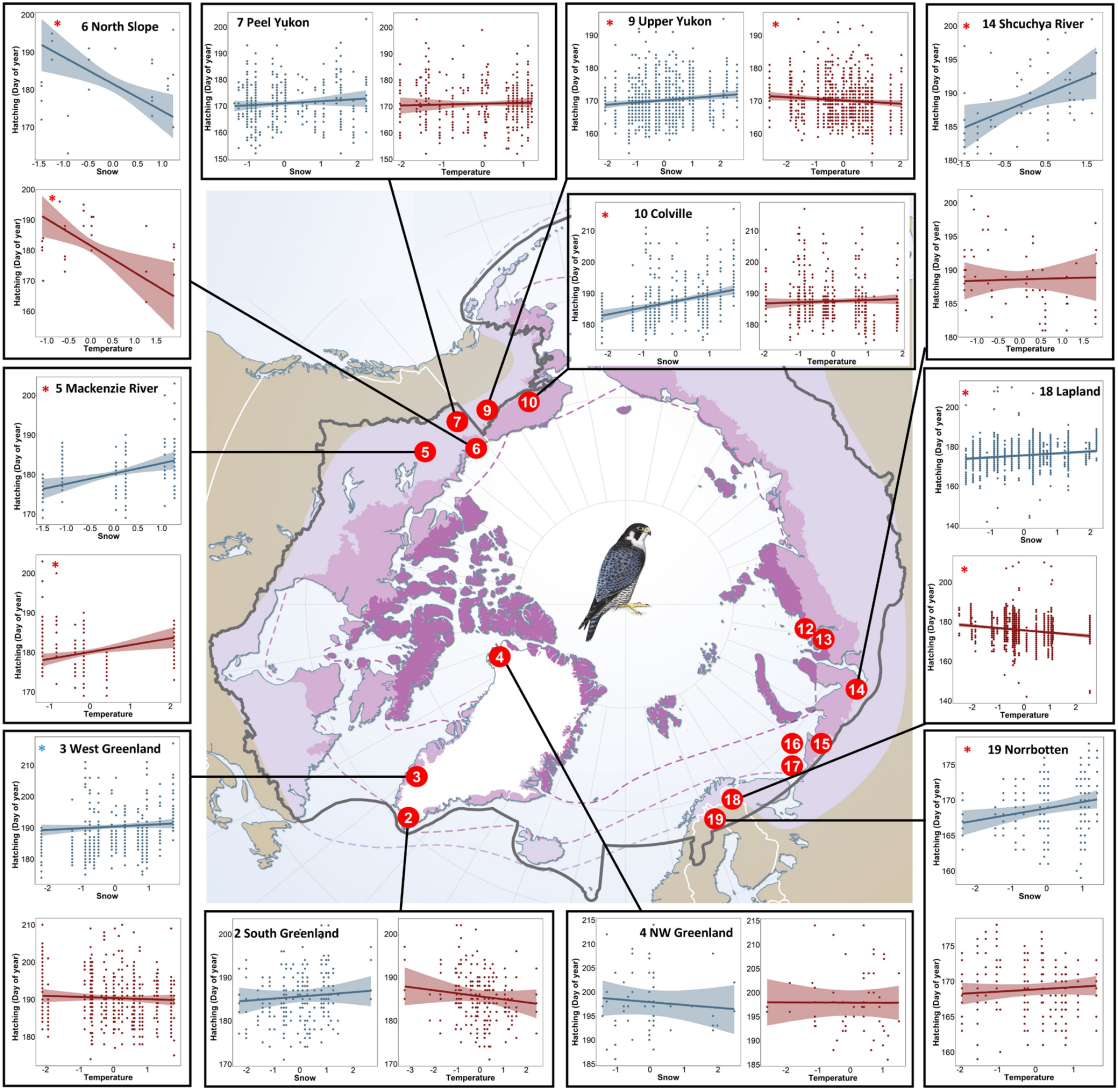
Univariate results for the effect of temperature (during egg‐laying stage) and first day of minimal snow cover (< 20%) on hatch dates among all long‐term study sites (*n* = 11) for peregrines (Analysis 2; see Section 2). The study period covers 1981–2022; note different axis units. Light purple refers to Sub Arctic, medium purple to Low Arctic, and dark purple to High Arctic as defined by Hohn and Jaakkola (2010) and the solid line is the boundary for Conservation of Arctic Flora and Fauna (CAFF) biodiversity monitoring plans (Christensen et al. 2013). Red stars indicate effects where 95% credible intervals did not overlap zero, reflecting the strongest evidence for an effect, while blue stars indicate effects with moderate directional support where credible intervals overlapped zero but probability of direction values suggested consistent directional trends.

**FIGURE 5:**
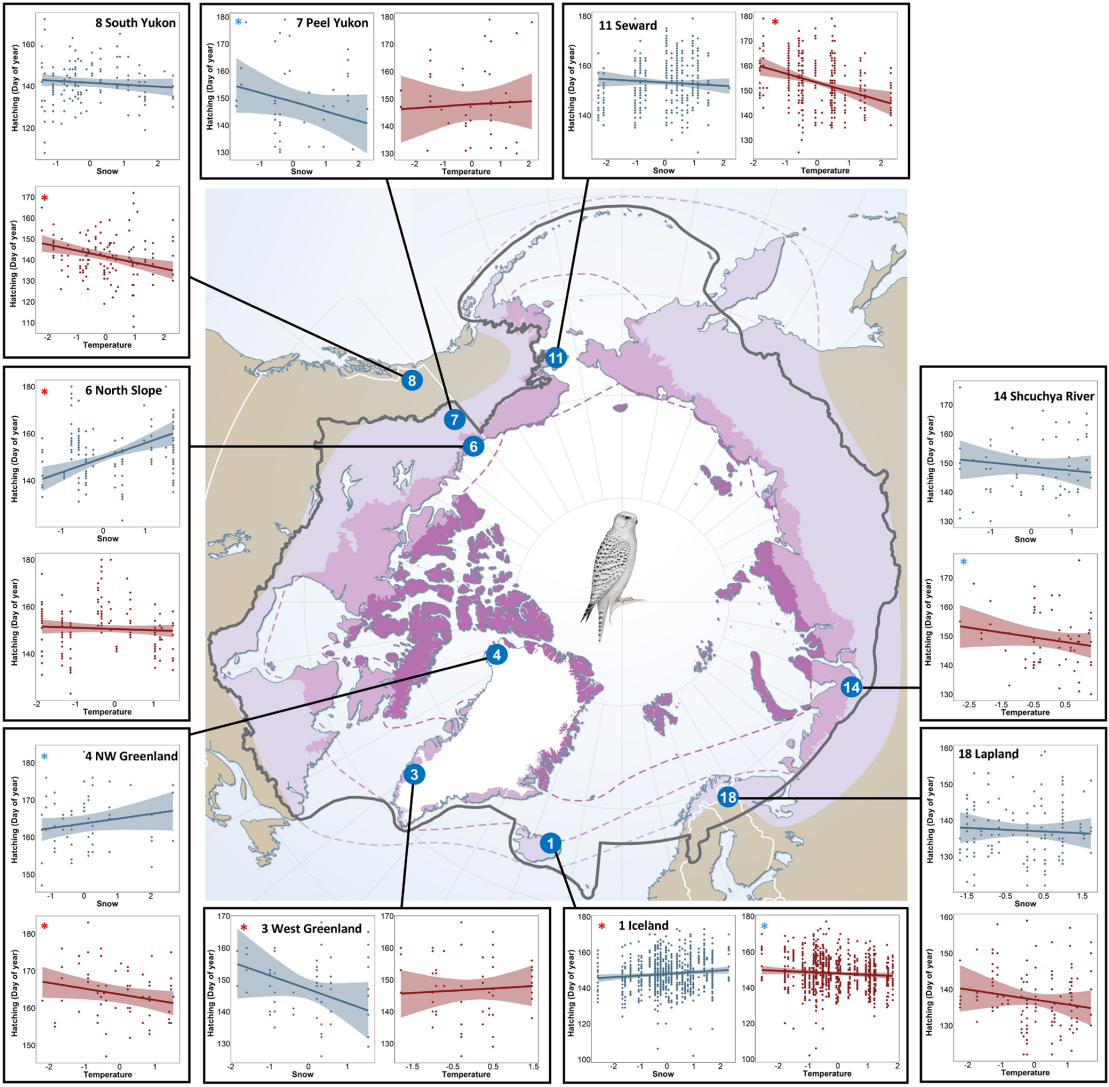
Univariate results for the effect of temperature (during egg‐laying stage) and first day of minimal snow cover (< 20%) on nestling hatch dates among all long‐term study sites (*n* = 9) for Gyrfalcons (Analysis 2; see Section 2). The study period covers 1981–2022. Light purple refers to Sub Arctic, medium purple to Low Arctic, and dark purple to High Arctic as defined by Hohn and Jaakkola (2010) and the solid line is the boundary for Conservation of Arctic Flora and Fauna (CAFF) biodiversity monitoring plans (Christensen et al. 2013). Red stars indicate effects where 95% credible intervals did not overlap zero, reflecting the strongest evidence for an effect, while blue stars indicate effects with moderate directional support where credible intervals overlapped zero but probability of direction values suggested consistent directional trends.

For environmental covariates, we found consistent evidence that later snowmelt was associated with delayed hatch dates in peregrines, with seven of 11 monitoring sites showing this relationship (Table 1; Figure 4). Of these, six study sites showed strong support (CrI not overlapping zero), while one site showed moderate directional support, with the posterior distribution aligned with delayed hatch dates. One site with a small sample size (North Slope, CA, site 6, *n* = 40) showed the opposite pattern (Table 1; Figure 4). A similar relationship between later snowmelt and delayed hatch dates was observed at three of the nine gyrfalcon monitoring sites. Two of these sites showed strong support (CrI not overlapping zero), while one showed moderate directional support, with the posterior distribution aligned with delayed hatch dates. In contrast, one Low Arctic (West Greenland, site 3) and one Sub Arctic site (Peel Yukon, CA, site 7) showed the opposite pattern, although estimates overlapped zero and provided only directional evidence for earlier hatch dates (Table 1; Figure 5). At the regional level, delayed snowmelt was linked to later hatch dates for peregrines in the Sub Arctic (*β* = 1.20, 95% CrI = 0.92, 1.47, PD = 100%), the Low Arctic (*β* = 1.01, 95% CrI = 0.46, 1.57, PD = 99.98%) and pooled data across all Arctic regions (Table 2; Figure 3). In contrast, for the gyrfalcon, we did not find evidence that snowmelt influenced nestling hatch dates at the regional level (Table 2; Figure 3).

Furthermore, we found that warmer temperatures during the egg‐laying stage were linked to earlier hatch dates in three of the 11 peregrine falcon study sites and five of the nine gyrfalcon study sites (Table 1; Figures 4 and 5). Among gyrfalcons, most sites showed strong support for this relationship, while two sites showed moderate directional support, with posterior distributions largely aligned with earlier hatch dates (Table 1; Figures 4 and 5). In the Sub Arctic, Low Arctic, and pooled data across all Arctic regions, warmer temperatures during the egg‐laying stage led to earlier hatch dates for both species (Table 2; Figure 3).

The analysis of vegetation greenness on hatch dates revealed that greater NDVI levels corresponded with earlier hatch dates at three of the 11 peregrine study sites and five of the nine gyrfalcon sites (Table 1). Among peregrines, two sites showed strong support and one showed directional support, while among gyrfalcons, three sites showed strong support and two showed directional support (Table 1). Lastly, at the regional level, increased levels of summer NDVI were linked to earlier hatch dates for gyrfalcons in the Sub Arctic (*β* = −1.86, 95% CrI = −2.80, −0.91, PD = 100%), and across all Arctic regions (*β* = −1.23, 95% CrI = −2.02, −0.45, PD = 99.91%), both showing strong support, while the Low Arctic showed a similar pattern, although estimates overlapped zero and provided only directional evidence (*β* = −0.82, 95% CrI = −1.91, 0.25, PD = 93.26%; Table 1; Figure 5). This pattern of earlier hatch dates with higher NDVI was also observed for peregrines, but only in the Sub Arctic (*β* = −0.50, 95% CrI = −0.78, −0.22, PD = 99.97%) and for pooled data across all Arctic regions (Table 2; Figure 3).

### 3.3 Effects of Year and Environmental Covariates on Productivity Across Arctic Regions

**FIGURE 6:**
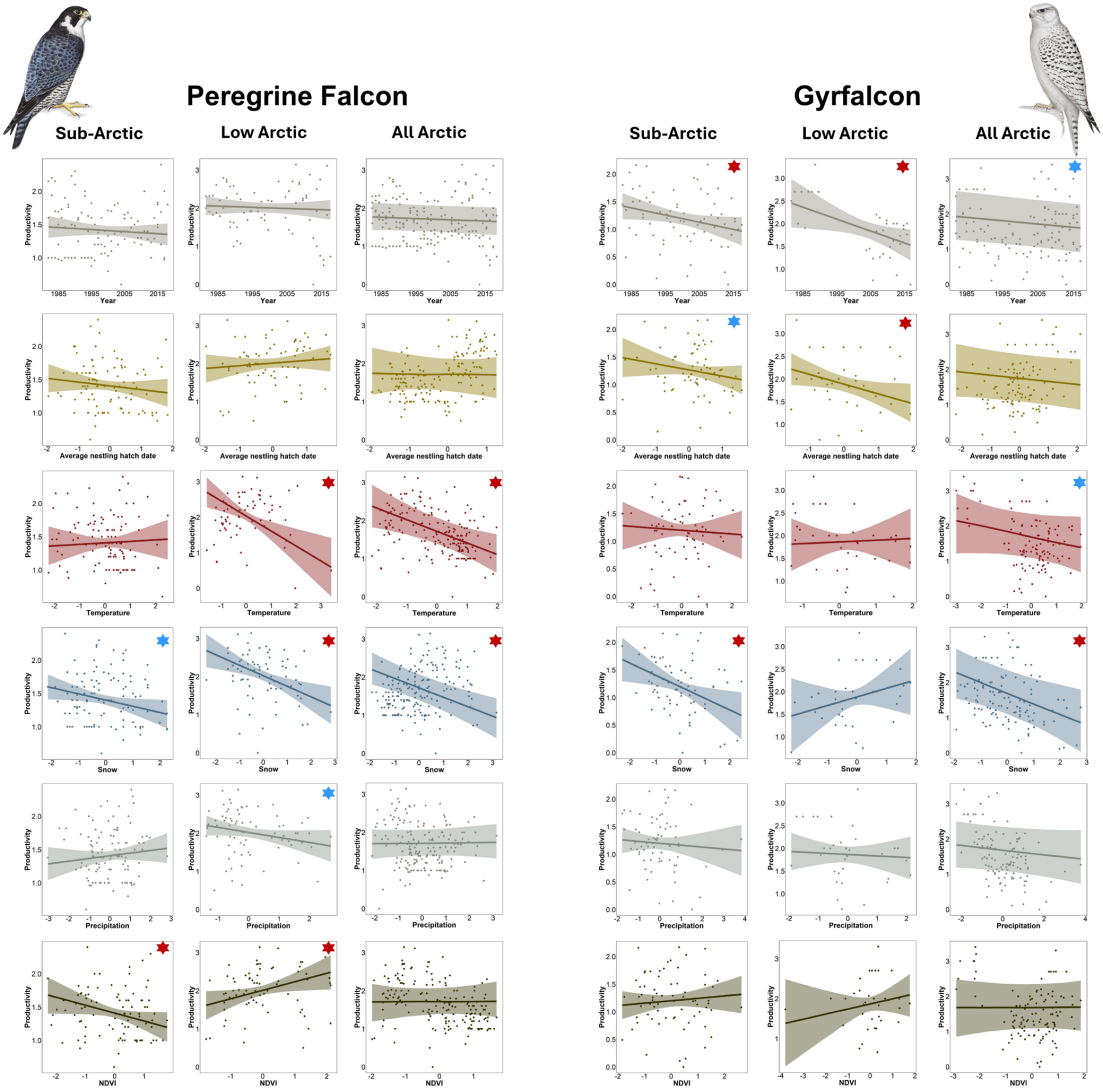
Univariate results for the effect of environmental covariates on falcon productivity among Arctic regions (Analysis 4; see Section 2). The study period covers 1981–2018 for peregrines and 1981–2017 for gyrfalcons. Red stars indicate effects where 95% credible intervals did not overlap zero, reflecting the strongest evidence for an effect, while blue stars indicate effects with moderate directional support where credible intervals overlapped zero but probability of direction values suggested consistent directional trends.

We had a total of 171 yearly estimates of productivity for peregrines at seven study sites (*n* = 98 Sub Arctic, *n* = 73 Low Arctic), and a total of 109 yearly estimates of productivity for gyrfalcons at six study sites (*n* = 66 Sub Arctic, *n* = 33 Low Arctic, *n* = 10 High Arctic). A slight downward trend in gyrfalcon productivity over time was observed across Arctic regions, with posterior estimates generally indicating a negative year effect, though support was moderate across pooled data (Table 3; Figure 6), while we did not observe an effect of year for peregrines (Table 3; Figure 6). For peregrines, we did not find an effect of earlier hatch dates on reproductive success (Table 3; Figure 6). However, for gyrfalcons, earlier nestling hatch dates were associated with increased productivity across Arctic regions, with strong support in the Low Arctic (*β* = −0.22, 95% CrI = −0.42, −0.01, PD = 98.16%) and more moderate, directional support in the Sub Arctic (*β* = −0.11, 95% CrI = −0.28, 0.05, PD = 91.31%). Pooled estimates indicated a similar trend, although support was weaker and estimates overlapped zero (Table 3; Figure 6).

**TABLE 3:** Univariate results for the effect of environmental covariates on productivity among Arctic regions for peregrines and gyrfalcons.

Species	Region	Intercept	Year		Average hatch date		Snow		Temperature		Precipitation		NDVI	
		*β* (95% CrI)	*β* (95% CrI)	PD (%)	*β* (95% CrI)	PD (%)	*β* (95% CrI)	PD (%)	*β* (95% CrI)	PD (%)	*β* (95% CrI)	PD (%)	*β* (95% CrI)	PD (%)
Peregrine Falcon	Sub Arctic	1.41 (1.33, 1.49)	−0.05 (−0.15, 0.06)	80.96	−0.06 (−0.16, 0.05)	85.39	**−0.08 (−0.17, 0.02)**	94.16	0.03 (−0.09, 0.15)	70.13	0.03 (−0.05, 0.11)	77.85	−0.14 (−0.26, −0.02)*	98.70
	Low Arctic	2.06 (1.93, 2.19)	−0.01 (−0.18, 0.15)	56.00	0.07 (−0.11, 0.25)	77.70	−0.28 (−0.46, −0.11)*	99.93	−0.42 (−0.66, −0.17)*	99.94	**−0.10 (−0.27, 0.06)**	89.18	0.21 (0.01, 0.41)*	97.85
	Pooled	1.72 (1.28, 2.09)	0.00 (−0.09, 0.09)	51.72	−0.03 (−0.27, 0.25)	57.96	−0.22 (−0.35, −0.08)*	99.87	−0.33 (−0.54, −0.12)*	99.84	0.00 (−0.10, 0.09)	53.40	0.12 (−0.11, 0.34)	84.79
Gyrfalcon	Sub Arctic	1.27 (1.17, 1.37)	−0.13 (−0.25, −0.01)*	98.43	**−0.11 (−0.28, 0.05)**	91.31	−0.18 (−0.34, −0.03)*	99.05	0.00 (−0.16, 0.17)	51.55	−0.03 (−0.13, 0.08)	69.32	0.03 (−0.10, 0.16)	66.90
	Low Arctic	2.01 (1.83, 2.19)	−0.43 (−0.74, −0.11)*	99.43	−0.22 (−0.42, −0.01)*	98.16	0.13 (−0.23, 0.49)	76.49	0.00 (−0.40, 0.40)	51.27	−0.05 (−0.25, 0.15)	69.75	0.11 (−0.16, 0.38)	79.09
	Pooled	1.76 (1.11, 2.45)	**−0.11 (−0.23, 0.01)**	96.99	−0.09 (−0.24, 0.06)	88.03	−0.32 (−0.53, −0.11)*	99.91	**−0.15 (−0.37, 0.06)**	91.78	−0.05 (−0.16, 0.05)	85.00	0.00 (−0.17, 0.18)	53.06

*Note:* The study period covers 1981–2018 for peregrines and 1981–2017 for gyrfalcons (Analysis 4; see Section 2). We report beta estimates (*β*) as posterior medians, 95% credible intervals, and probability of direction (PD). Effects where credible intervals did not overlap zero were interpreted as having the strongest directional support. Effects where credible intervals overlapped zero but PD was ≥ 89% were interpreted as having moderate directional support. Both are highlighted in bold, with effects having the strongest directional support additionally marked with an asterisk. All covariates were standardized prior to analysis.

Timing of snowmelt was linked to productivity for both species, with delayed snowmelt associated with reduced average number of young (Table 3; Figure 6). For peregrines, this pattern showed strong support in the Low Arctic (*β* = −0.28, 95% CrI = −0.46, −0.11, PD = 99.93%) and more moderate, directional support in the Sub Arctic (*β* = −0.08, 95% CrI = −0.17, 0.02, PD = 94.16%). For gyrfalcons, a similar relationship was evident in the Sub Arctic and in pooled analyses (Table 3; Figure 6). Additionally, higher summer temperatures corresponded with lower productivity in both species, with strong support in the Low Arctic for peregrines (*β* = −0.42, 95% CrI = −0.66, −0.17, PD = 99.94%). A similar negative relationship was observed in pooled analyses for both species, although support for gyrfalcons was more moderate and primarily directional, with posterior estimates largely aligned with reduced productivity (Table 3; Figure 6).

We found little evidence of an association between summer precipitation and gyrfalcon productivity (Table 3; Figure 6). In contrast, for peregrines in the Low Arctic, greater precipitation was associated with reduced productivity, although support was moderate and primarily directional (*β* = −0.10, 95% CrI = −0.27, 0.06, PD = 89.18%). Lastly, we found no impact of summer NDVI on gyrfalcon productivity (Table 3; Figure 6) but, for peregrine falcons, we observed mixed results, with both positive and negative effects on productivity (Table 3; Figure 6).

## 4 Discussion

Firstly, it should be noted that the power of our analyses is limited because the environmental data are averaged at the monitoring site level and cannot account for variation in local conditions at individual falcon territories. In addition, our results may be somewhat biased as the surveys in much of the monitoring areas and years have been conducted during the nestling stage; this may overestimate productivity and—since the timing of breeding is determined by back‐calculating from the age of the young—it could exclude the laying dates of early nesters that may have failed due to severe weather (Zuberogoitia et al. 2018). Despite these limitations, our study identified a range of interactions between breeding phenology, weather parameters, and productivity across the Arctic regions.

### 4.1 Breeding Phenology

We confirmed that, on average, gyrfalcons hatched around 34 days earlier than peregrines across each Arctic region and as expected, hatch dates were the earliest in the Sub Arctic and latest in the High Arctic for both species. The resident gyrfalcon, relying on resident prey, in particular adult ptarmigan, can initiate laying well before the migrant peregrine who is dependent largely on migrant prey species. We also found that while nestling hatch dates changed over time at many study sites, not all sites shifted consistently in the same direction, and there was considerable year‐to‐year variability at each site (Figure 2). Hatching occurred earlier over time at 37% of the study sites for peregrine and 55% of the gyrfalcon sites, but later breeding was noted in 19% of the peregrine and 11% of the gyrfalcon sites. However, the change in breeding season is modest in most areas. When pooled at the regional level, hatch dates occurred earlier over time in the Low Arctic for both species (peregrine 0.41 days/decade earlier, gyrfalcon 1.62 days/decade earlier), and later in the Sub Arctic for gyrfalcon (0.42 days/decade later). Maximum changes at the specific monitoring site level were up to 2.81 days/decade earlier (site 7, Peel Yukon) or later (site 4, Northwest Greenland) for peregrines, but for gyrfalcons it was up to 9.30 days/decade earlier (site 3, West Greenland) with the extreme of 21.01 days/decade earlier (site 7, Peel Yukon), albeit based on a very small sample size (Figure 2).

Interestingly, earlier hatching occurred in parallel for both falcon species in some study sites, like sites 3 (Central west Greenland) and 7 (Peel Yukon), despite their distance from each other, while at site 4 (Northwest Greenland), the trends differed between species (see Figure 2). At the same time, despite their proximity in northwestern North America, differing phenological trends were observed at peregrine sites 7 (Peel Yukon) and 9 (Upper Yukon), a pattern also noted for gyrfalcons at site 8 (South Yukon) which differed from nearby study sites 6 (North Slope), 7 (Peel Yukon), and 11 (Seward Peninsula, Figure 2). Variations in breeding phenology among populations that are near one another has also been identified in the blue tit ( *Cyanistes caeruleus* ; Porlier et al. 2012). While our data does not examine the effects of individual laying decisions on nest‐level reproductive outcomes, we propose that local selection pressures favoring either earlier or later hatching dates may be responsible for the inconsistent patterns we observed across spatially adjacent study sites within each species (Porlier et al. 2012; Le Vaillant et al. 2021; Halupka et al. 2023). For instance, snow accumulation in early spring can limit access to quality nesting sites, restricting high‐quality territories to more dominant and aggressive individuals. While aggression is often linked to earlier laying and better territory acquisition in raptors (Kontiainen et al. 2009), studies on Arctic peregrines (Gulotta and Mathot 2022) and other avian species (Betini and Norris 2012) suggest its fitness benefits vary with environmental conditions, indicating that individual level behavior (i.e., animal personality) may influence phenology and ultimately fitness in context‐dependent ways (Porlier et al. 2012; Abbey‐Lee and Dingemanse 2019; Harris et al. 2020; Le Vaillant et al. 2021; Zabala et al. 2022).

Our data also suggests that peregrines and gyrfalcons exhibit varied phenological responses within the same study areas, highlighting the need for additional research to explore how environmental factors and individual behavior differently influence the breeding ecology of these two species. For monitoring site 10, Colville River, Swem and Matz (2018) reported later hatching dates, but in this reanalysis with a longer time series available, the trend was reversed to show earlier hatching dates. These observations accentuate the importance of long‐term monitoring, but also that the changes in phenology in peregrines are modest and with large interannual variability. In resident peregrine populations outside the Arctic, a tendency toward delayed incubation onset has been attributed to more adverse weather conditions in the pre‐laying stage, including heavy rainfall events associated with strong winds and low temperatures which may also cause systematic loss of early clutches (Zuberogoitia et al. 2018; Sarà et al. 2022). Data on such short‐term weather variability was not available for our analysis. Other possible sources that could be influencing variability in hatch dates include loss of mate, disturbance during courtship, and age of females (Zuberogoitia et al. 2009, 2018), factors that should be given more focus in future detailed studies in the Arctic.

### 4.2 Effects of Environmental Covariates on Breeding Phenology

In northern landscapes, the brief summers and the falcons' extensive breeding cycles heighten reproductive costs, emphasizing the need for reliable cues for nest initiation due to their dependence on consistent prey supply (Lamarre et al. 2017). Studies indicate that female peregrines assess male provisioning to gauge prey availability, impacting clutch initiation and reproductive success (Cade 1960; Wrege and Cade 1977; Olsen et al. 1998; Lamarre et al. 2017), while environmental factors like delayed snowmelt can further disrupt nesting and decrease productivity (Cade 1960; Poole and Bromley 1988; Bradley et al. 1997; Lamarre et al. 2017). Our findings suggest that snowmelt timing is likely an important environmental cue for assessing resource availability in peregrines, with earlier snowmelt linked to earlier nestling hatch dates and increased productivity across all Arctic regions, whereas delayed snowmelt not only postpones hatch dates but also reduces productivity, as also noted in a range of other Arctic species (Ratcliffe 1993; Bradley et al. 1997; Madsen et al. 2007; Robinson et al. 2014; Boelman et al. 2017). Similarly, delayed snowmelt decreased productivity in gyrfalcons, indicating that such delays could impact prey availability and hunting efficiency for both falcon species by changing the breeding phenology of prey and thus decreasing the availability of naive prey at critical periods during brood rearing and post‐fledging (Durant et al. 2007; Both et al. 2009; Grabowski et al. 2013; Liebezeit et al. 2014; Kleiven et al. 2025). For the gyrfalcon, however, delayed snow melt can also affect access to nesting ledges (Henderson et al. 2021; Platt 1976). Consequently, the long‐term trend of earlier snowmelt across the Arctic (see Figure S1 for trends at our study sites) seems to be an important driver of the earlier breeding seasons observed at most study sites for peregrines. Although gyrfalcons' breeding phenology remains largely unaffected by snowmelt (except for a few study sites), the breeding phenology of prey for both gyrfalcons and peregrines is likely shifting with earlier snowmelt (Durant et al. 2007; Both et al. 2009; Grabowski et al. 2013; Liebezeit et al. 2014), potentially impacting the timing of peak prey availability.

Snowmelt is, of course, also tightly coupled to spring temperature, and our study also shows that warmer spring temperatures lead to earlier nestling hatch dates. In the Sub Arctic and Low Arctic, higher temperatures were clearly linked to earlier hatch dates for both falcon species. Similarly, Dickey et al. (2008) found that warmer springs resulted in earlier clutch initiation dates for greater snow geese ( *Chen caerulescens atlantica* ) in High Arctic Nunavut, and our study reaffirms this phenomenon across the circumpolar Arctic (Dickey et al. 2008; Boelman et al. 2017; Sauve et al. 2019). Moreover, numerous studies suggest that long‐distance migrants typically respond less to climate warming than resident or short‐distance migrants, which are more sensitive to the cascading effects of temperature increases in the breeding area (Kluen et al. 2017; Usui et al. 2017; Samplonius et al. 2018; Søraker et al. 2022). While both peregrines (Sub Arctic—0.23 days earlier/1°C increase; Low Arctic—0.24 days earlier/1°C increase) and gyrfalcons advanced hatch dates with rising temperatures, the response was stronger in resident gyrfalcons across all Arctic regions (Sub Arctic—0.75 days earlier/1°C increase; Low Arctic—0.97 days earlier/1°C increase; Pooled—0.97 days earlier/1°C increase). In addition, we also demonstrate that greater NDVI levels (vegetation greenness) during the hatching stage, indicative of an earlier spring, correlated with earlier nestling hatch dates for gyrfalcons across all Arctic regions. Peregrines exhibited a similar pattern across all Arctic regions and in the Sub Arctic but not in the Low Arctic. Previous research has indicated that some Arctic breeding migratory birds, such as shorebirds, can adapt to changes in the timing of green‐up (Tavera et al. 2024), while others, like waterfowl, experience phenological mismatches (Clausen and Clausen 2013; Lameris et al. 2017, 2018). Our findings further support the idea that migratory species are less responsive to climate warming than residents, given that resident gyrfalcons adjusted more strongly to increases in both temperature and NDVI compared to migratory peregrines.

Furthermore, the difference in annual cycles between species may explain the contrasting adjustment of phenology over time. Resident and short‐distance migrating birds are expected to have annual schedules that respond more directly to environmental cues, while long‐distance migrants are expected to have endogenously controlled circannual rhythms that are less flexible (Åkesson and Helm 2020; Huffeldt 2021); i.e., in contrast to the resident gyrfalcons, peregrines are not exposed to the local weather cues at the breeding grounds while on their wintering grounds or during migration. Our data supports these expectations in that the long‐distance migrating peregrines advanced their phenology much less than the resident gyrfalcons. Our results align with other studies that have shown that long‐distance migrants possess more fixed annual schedules and are less likely to align with the variations in spring green‐up or increasing spring temperatures compared to resident species (Kluen et al. 2017; Samplonius et al. 2018; Søraker et al. 2022). In particular, our finding that peregrines show modest long‐term advancement and strong annual coupling to local spring cues aligns with the idea that migrants may advance arrival mainly by traveling faster over years, yet remain constrained in year‐to‐year synchronization with snowmelt and in translating earlier arrival into earlier laying (Lameris et al. 2025).

Our circumpolar data, indicating temperature‐induced changes in breeding phenology, contrasts with Poole and Bromley (1988) who reported that, apart from an exceptional cold year, spring temperatures were not linked to the timing of gyrfalcon clutch initiation. Given the rising spring temperatures across Arctic regions (Table S4; Figure S1) and expected future rapid increase (Masson‐Delmotte et al. 2021), we anticipate that both peregrines and gyrfalcons will increasingly tend to nest earlier in response to warmer springs. Previous research on peregrine falcon spring migration has shown that greater temperatures correlate with earlier arrivals in the Arctic (Curk et al. 2020); however, explicit links between warmer temperatures, earlier arrivals, and individual laying decisions remain unexplored. Studies on pied flycatchers ( *Ficedula hypoleuca* ) have shown that warmer temperatures lead to earlier spring arrivals and breeding, both of which are under strong directional selection (Visser et al. 2015; Helm et al. 2019). In addition, Sarà et al. (2022) demonstrated that later incubation onset decreased productivity in Mediterranean peregrine populations, further supporting the notion that earlier breeding is advantageous across a range of falcon populations and climatic contexts. The mechanisms driving shifts toward earlier breeding are likely multifaceted and may include directional selection for earlier spring arrival and breeding as observed in other species (Gienapp et al. 2014; Visser et al. 2015; Bowers et al. 2016; Helm et al. 2019; Shave et al. 2019), phenotypic plasticity allowing individuals to adjust breeding timing in response to annual variation in environmental conditions (Dingemanse et al. 2010), or consistent among‐individual differences in behavioral traits (i.e., animal personality) that correlate with earlier or later breeding depending on environmental context (Abbey‐Lee and Dingemanse 2019; Harris et al. 2020). Disentangling these mechanisms in Arctic‐breeding peregrines represents an important direction for future research given the strong fitness consequences of breeding timing documented here and elsewhere (Sarà et al. 2022).

Beyond climate and plasticity, age‐related variation in breeding phenology may be an additional and underappreciated contributor to population‐level patterns. Zabala and Zuberogoitia (2015) and Zabala et al. (2022) showed that younger peregrine breeders tend to initiate incubation later in the season than older, more experienced individuals, and that this delay is associated with reduced reproductive output. If the age structure of Arctic breeding populations shifts over time in response to changing environmental conditions, this could interact with climate‐driven advances in spring phenology in ways that are difficult to predict without detailed longitudinal data. It therefore remains unknown whether individuals that consistently breed earlier do so because of age and experience, inherent individual quality, phenotypic plasticity, or some combination of these factors, and whether any of these pathways translate to consistently greater productivity across multiple seasons. Addressing these questions will require sustained monitoring of individually marked birds across multiple breeding seasons and represents an important priority for future research.

### 4.3 Effects of Breeding Phenology and Environmental Covariates on Productivity

In line with our hypothesis regarding productivity and earlier nest initiation, we found that earlier nestling hatch dates were associated with increased productivity for gyrfalcons, as also recorded specifically at site 8 (Seward Penisula—Henderson et al. 2021) in Alaska and site 3 (West Greenland—Burnham and Burnham 2011). Conversely, for peregrines, earlier hatch dates had no notable impact on productivity, which was unexpected. Many studies have demonstrated a relationship between earlier nesting and higher productivity (Perrins 1970; Verhulst and Nilsson 2008; Callery et al. 2022), including studies on gyrfalcons and peregrines (Nielsen 2003, Burnham and Burnham 2011; Henderson et al. 2021). Given the prolonged breeding cycle of gyrfalcons and the brief Arctic summers, it is understandable that earlier breeding should improve productivity. Earlier breeding likely ensures that the critical growth phases of nestlings, or independence of juveniles (Nielsen), coincide with the peak availability of food resources, which is similar to other bird species breeding in the Arctic (Dickey et al. 2008; Clausen and Clausen 2013; Doiron et al. 2015; Kwon et al. 2019; Saalfeld et al. 2019). In addition, our findings offer mixed evidence for our hypothesis that greater NDVI levels would increase falcon productivity. Specifically, we observed that for peregrines, greater NDVI levels improved productivity in the Low Arctic but decreased productivity in the Sub Arctic. However, for gyrfalcons, we found no association between NDVI levels and productivity. Similarly, precipitation appeared to have no impact on the productivity of gyrfalcons, but for peregrine falcons we found that greater levels of precipitation in the Low Arctic resulted in lower productivity, which supports findings from previous studies on peregrine falcons in the Low Arctic (Anctil et al. 2014; Carlzon et al. 2018). The gyrfalcon tends to select well protected nest ledges with overhang (Booms et al. 2020; Henderson et al. 2021) where offspring would be better shielded from heavy rain compared to peregrines, who often construct nests on exposed open ledges that leave nestlings vulnerable to heavy precipitation events (Falk et al. 1986; Anctil et al. 2014; White et al. 2024). Nestling age and the timing of heavy rain events could influence productivity (Zuberogoitia et al. 2018), which we were unable to account for given the coarseness of our environmental data.

Contrary to our hypothesis that warmer temperatures during the brood rearing stage would increase productivity, our findings suggest that higher summer temperatures decreased productivity for both falcon species. Although we did not expect this effect in the cool Arctic, heat stress has been described for both species (Booms et al. 2020; White et al. 2024), and Arctic birds are particularly sensitive to increased temperatures (Choy et al. 2021; O'Connor et al. 2024). Warmer temperatures at the beginning of the brood rearing stage can exacerbate heat stress in nestlings who are unable to regulate their body temperature (Levillain et al. 2025), especially those in nests without natural shade from overhangs. Studies on raptors, including peregrines, indicate that such conditions in warmer summers are linked to higher mortality rates among nestlings due to heat prostration (Nelson 1969; Beecham and Kochert 1975; Tomback and Murphy 1981; Kochert et al. 2019). While the gyrfalcon, a cold‐adapted Arctic specialist (Cade 2011), might be sensitive to warmer breeding seasons, the peregrine, which inhabits a range of climate zones, should generally be better adapted to handle heat and warm spells. However, local adaptation within peregrine populations could limit the individual flexibility needed to respond to a changing climate because local adaptation is known to limit fitness of individuals experiencing different environmental conditions than that of their origin (Bradshaw et al. 2004).

Field observations in another Arctic raptor, the rough‐legged buzzard ( *Buteo lagopus* ), show that during hot weather nestlings leave the nest to seek shade; sudden shifts to rain and cooling can then cause hypothermia and mortality if adults do not collect dispersed chicks back to the nest (Pokrovsky et al. 2012). Peregrines often nest on exposed ledges with limited shade, where mobile chicks can move beyond parental shelter, potentially increasing vulnerability to ‘heat‐then‐rain’ sequences. Gyrfalcons, by contrast, frequently use substantial stick nests and ledges with overhang that may reduce these risks. This may help explain why we detect a stronger negative effect of summer temperature (and precipitation in the Low Arctic) on peregrine productivity, while gyrfalcon productivity shows no consistent temperature effect. Nevertheless, heat stress of gyrfalcon nestlings has been noted in Canada and Northeast Greenland (Platt 1976; Fletcher and Webby 1977; Poole and Bromley 1988); in West Greenland gyrfalcon nestlings have been found dead below nests because they appeared to have had to jump out of the nest because it was too hot (K. Burnham, pers. observations).

We speculate that both species could face challenges if, early in the spring, they choose their nest sites based on a snow‐free, warm, and dry ledge (typically south‐facing for Arctic peregrines; White et al. 2024; Falk et al. 1986), which initially seems advantageous. Choosing such nest sites could become problematic, as they may become heat traps when summer temperatures rise. This could be particularly concerning for peregrines, which breed about a month later than gyrfalcons, thus exposing their small young to the peak midsummer solar irradiation and in‐nest temperatures. If these assumptions hold true, nestlings of both species could face greater rates of mortality from heat prostration than previously thought.

## 5 Conclusion

We acknowledge that our environmental data, averaged at the monitoring site level, are crude and cannot account for variation in local conditions at individual falcon territories. This limits the power of our analyses of linkages to falcon phenology and productivity, so more specific research would be warranted. In particular, the average values for weather variables mask the increasing variability which may impact productivity, e.g., occurrence of a single significant storm or heat wave that can kill nestlings. We advise that more detailed sampling should be conducted at long‐term study sites to help disentangle the factors affecting the breeding phenology and productivity of both species, to support conservation efforts. We recommend holistic monitoring of individual territories and falcons over multiple breeding seasons to assess patterns of selection to infer whether directional selection for earlier breeding is occurring in either species, while at the same time accounting for individual level plasticity to environmental change (Dingemanse et al. 2010). Likewise, linking the breeding phenology of falcons with those of their prey is essential to understand the potential effects of trophic mismatches on productivity, which has been demonstrated in other avian species breeding in the Arctic (Doiron et al. 2015; Ross et al. 2017, 2018). Due to lack of data from the High Arctic, we were unable to evaluate the impact of environmental conditions on productivity for both falcon species in that region, and enhanced monitoring efforts in the High Arctic are much needed (Franke et al. 2020). With increasing warming of the Arctic, the gyrfalcon populations in the High Arctic may be the most important as they may well be the only populations left in 50–100 years.

In conclusion, our findings demonstrate that nestling hatch dates for both species are changing over time, with environmental conditions like snowmelt and temperature playing a significant role in influencing not only the timing of nestling hatch dates but also productivity. Given the rising spring temperatures across Arctic regions (including our sampling areas, Table S4, Figure S1) and the expected future rapid increase (Masson‐Delmotte et al. 2021), we would anticipate that both peregrines and gyrfalcons will increasingly tend to nest earlier in response to warmer springs. However, in line with Schmidt et al. (2023), climate change may not only influence a direction of change; the increased variability in weather patterns and associated environmental conditions may also induce more variable responses in the falcons' breeding ecology, both in timing of breeding and in productivity. Hence, different populations may respond differently to climatic challenges, adjusting phenology either earlier or later depending on the context (Porlier et al. 2012; Abbey‐Lee and Dingemanse 2019; Harris et al. 2020; Le Vaillant et al. 2021; Zabala et al. 2022), affecting population parameters such as productivity and possibly adult and juvenile survival (Zuberogoitia et al. 2009, 2018). Continued coordinated circumarctic studies and analyses are needed to identify the variable responses of the top predators in the fast‐warming Arctic.

## Author Contributions

**Nick A. Gulotta:** conceptualization, investigation, writing – original draft, methodology, visualization, writing – review and editing, formal analysis, data curation, project administration. **Knud Falk:** conceptualization, investigation, writing – review and editing, data curation, methodology, writing – original draft, visualization, project administration. **Skip Ambrose:** data curation, investigation, writing – review and editing. **Nicholas W. Bakner:** writing – review and editing. **Peter J. Bente:** writing – review and editing, data curation, investigation. **Travis L. Booms:** writing – review and editing, data curation, investigation. **Kurt K. Burnham:** writing – review and editing, data curation, investigation. **Suzanne Carrière:** writing – review and editing, data curation, investigation. **Michael T. Henderson:** writing – review and editing, data curation, investigation. **Sergey Kharitonov:** writing – review and editing, data curation, investigation. **Alexander V. Kondratyev:** writing – review and editing, data curation, investigation. **Olga Kulikova:** writing – review and editing, data curation, investigation. **Peter Lindberg:** writing – review and editing, data curation, investigation. **Berth‐Ove Lindström:** writing – review and editing, data curation, investigation. **Svetlana Mechnikova:** writing – review and editing, data curation, investigation. **Dave Mossop:** writing – review and editing, data curation, investigation. **Søren Møller:** writing – review and editing, data curation, investigation. **Ólafur K. Nielsen:** writing – review and editing, data curation, investigation. **Kent Nilsson:** writing – review and editing, data curation, investigation. **Tuomo Ollila:** writing – review and editing, data curation, investigation. **Sture Orrhult:** writing – review and editing, data curation, investigation. **Ivan Pokrovsky:** writing – review and editing, data curation, investigation. **Marco Restani:** writing – review and editing, data curation, investigation. **Bryce W. Robinson:** writing – review and editing, data curation, investigation. **Robert N. Rosenfield:** investigation, writing – review and editing, data curation. **Ted Swem:** writing – review and editing, data curation, investigation. **Patrick H. Wightman:** writing – review and editing. **Nicholas P. Huffeldt:** writing – review and editing.

## Funding

This work was supported by The Danish Environmental Protection Agency 15. Juni Fonden. Bodil Pedersen Fonden Aage V. Jensens Fonde. U.S. Army Edgewood Research, Development, and Engineering Center, Aberdeen Proving Ground, Maryland. Offield Family Foundation. Wolf Creek Charitable Trust. National Geographic Society. Peregrine Fund. Natural Science Institute of Iceland.

## Conflicts of Interest

The authors declare no conflicts of interest.

## Supporting information


**Table S1:** Characteristics of Arctic gyrfalcon and peregrine falcon monitoring sites included in analyses.
**Table S2:** Model descriptions used in each analysis within the main text for both gyrfalcon and peregrine falcon hatch date and productivity data.
**Table S3:** Univariate results for sources of variation in hatch dates among Arctic regions for peregrine falcons and gyrfalcons. The study period covers 1967–2022 for peregrine falcons and 1973–2022 for gyrfalcons. We fit nestling hatch dates as the response variable, with fixed effects of Arctic region, species, an interaction between Arctic region and species, and random effects of site and year. We report beta estimates (*β*) as posterior medians, 95% credible intervals, and probability of direction (PD). Effects where credible intervals did not overlap zero were interpreted as having the strongest directional support. Effects where credible intervals overlapped zero but PD was ≥ 89% were interpreted as having moderate directional support.
**Table S4:** Univariate results for the effect of year on each environmental covariates among Arctic regions. The study period covers 1981–2022. We fit environmental covariates as the response variable and a fixed effects of year. We scaled and centered all environmental covariates and year prior to analysis and report beta estimates (*β*) as posterior medians, 95% credible intervals, and probability of direction (PD). Effects where credible intervals did not overlap zero were interpreted as having the strongest directional support. Effects where credible intervals overlapped zero but PD was ≥ 89% were interpreted as having moderate directional support.
**Table S5:** Variance inflation factor (VIF) results for environmental covariates included in models of hatch date across all long‐term monitoring sites for peregrines (*n* = 11) and gyrfalcons (*n* = 9) from 1981 to 2022. This table corresponds to the model results presented in Table 1 of the main text.
**Table S6:** Variance inflation factor (VIF) results for year and environmental covariates included in univariate models of hatch date across Arctic regions for peregrines and gyrfalcons from 1981 to 2022.
**Table S7:** Variance inflation factor (VIF) results for environmental covariates included in univariate models of productivity across Arctic regions for peregrines (1981–2018) and gyrfalcons (1981–2017).
**Figure S1:** Changes in environmental parameters across years 1982–2022 at falcon monitoring sites in different zones of the Arctic. Each dot contained within each plot represents environmental data from a specific monitoring site and year.

## Data Availability

All R‐scripts and data required to replicate the analyses presented in this manuscript are available on Zenodo: https://doi.org/10.5281/zenodo.18024718 (Gulotta et al. 2025).
