# Supplementary material for: Resident and Migratory Falcons' Breeding Phenology and Productivity Respond Differently to Weather and Climate Change Across the Arctic
Source: Glob Chang Biol. 2026 Aug 5;32(8):e71022. doi: 10.1111/gcb.71022 (PMC13439350; doi:10.1111/gcb.71022)
Supplement: Supplementary file 1 [file GCB-32-e71022-s001.docx]

**SUPPLEMENTARY MATERIAL**

**Resident and migratory falcons breeding phenology and productivity respond differently to weather and climate change across the Arctic**

Nick A. Gulotta^1^, Knud Falk, Skip Ambrose, Nicholas W. Bakner, Peter J. Bente, Travis L. Booms, Kurt K. Burnham, Suzanne Carrière, Michael Henderson, Sergey Kharitonov, Alexander V. Kondratyev, Olga Kulikova, Peter Lindberg, Berth-Ove Lindström, Svetlana Mechnikova, Dave Mossop, Søren Møller, Ólafur K. Nielsen, Kent Nilsson, Tuomo Ollila, Sture Orrhult, Ivan Pokrovsky, Marco Restani, Bryce W. Robinson, Robert N. Rosenfield, Ted Swem, Patrick H. Wightman, Nicholas P. Huffeldt

**Table S1**. Characteristics of Arctic gyrfalcon and peregrine falcon monitoring sites included in analyses

| **Site** ^a^ | **Species** | **Country** | **Monitoring site** | **Coordinates** | | **Arctic region** | **Landscape type** | **Elevation (m)** | **Survey area extent** |  |
| --- | --- | --- | --- | --- | --- | --- | --- | --- | --- | --- |
| 1 | GYRF | Iceland | Northeast Iceland | 65.4 | -17.0 | Sub | coastal/inland | 0-800 | 5300 km^2^ |  |
| 2 | PEFA | Greenland | South Greenland | 61.0 | -45.5 | Low | inland/fjord | 0 - 1780 | 3000 km^2^ |  |
| 3 | PEFA / GYRF | Greenland | Central west Greenland | 67.0 | -50.7 | Low | inland | 0-400 | 6500 km^2^ |  |
| 4 | PEFA / GYRF | Greenland | Northwest Greenland | 77.0 | -68.6 | High | coastal | 0 -300 | 750 km |  |
| 5 | PEFA | Canada | Mackenzie River | 66.0 | -125.0 | Sub | inland, riparian | 200-500 | 800 km |  |
| 6 | PEFA / GYRF | Canada | North Slope Yukon | 69.4 | -139.0 | Low | coastal/inland | 1-400 | 3600 km^2^ |  |
| 7 | PEFA / GYRF | Canada | Yukon/Peel Rivers | 63.7 | -139.7 | Sub | inland, riparian | 200-300 | 600 km |  |
| 8 | GYRF | Canada | South Yukon | 60.0 | -136.5 | Sub | inland | 600-1000 | 200 km^2^ |  |
| 9 | PEFA | USA | Upper Yukon River | 65.0 | -143.0 | Sub | inland, riparian | 170-300 | 265 km |  |
| 10 | PEFA | USA | Colville River | 69.5 | -151.6 | Low | inland, riparian |  | 345 km |  |
| 11 | GYRF | USA | Seward Peninsula | 65.2 | -165.2 | Low | coastal/inland | 0-1000 | 14150 km^2^ |  |
| 12,13 | PEFA | Russia | Taimyr, Agapa/Meduza | 71.0 | 87.0 | High | coastal/inland | 0-300 | 350 km |  |
| 14 | PEFA / GYRF | Russia | Schuchya River | 67.5 | 69.2 | Low | inland/riparian | 0-200 | 1300 km^2^ |  |
| 15 | PEFA | Russia | Nenetskiy Ridge | 68.3 | 53.2 | Low | coastal/inland | 0 – 185 | 100 km^2^ |  |
| 16 | PEFA | Russia | Kolguev Island | 69.1 | 49.0 | Low | inland | 20 -185 | 500 km^2^ |  |
| 17 | PEFA | Russia | Kanin Peninsula | 68.0 | 45.0 | Low | coastal/inland | 0-100 |  |  |
| 18 | PEFA / GYRF | Finland | Lapland | 69.0 | 27.2 | Sub | inland | 100-1000 | 15000 km^2^ |  |
| 19 | PEFA | Sweden | Norrbotten | 67.0 | 22.0 | Sub | inland | 0-1000 | 78000 km^2^ |  |
| a: See Figure 2 | | | | | | | | |  | |

| **Model** | **Data used** | **Species** | **Fixed effects** | **Random effects** | **Error distribution** | **Prior** | **Analysis #^a^** |
| --- | --- | --- | --- | --- | --- | --- | --- |
| Breeding phenology | Regional data^1^ | Peregrine falcon, gyrfalcon | Region*Species + Region | Year, Site | Gaussian | Default in brms package (uninformative) | Analysis 1 |
| Breeding phenology + environmental covariates | Site level data^4^ | Peregrine falcon | Year + Snow + Spring temperature + NDVI | Breeding territory | Gaussian | Default in brms package (uninformative) | Analysis 2 |
| Breeding phenology + environmental covariates | Site level data^5^ | Gyrfalcon | Year + Snow + Spring temperature + NDVI | Breeding territory | Gaussian | Default in brms package (uninformative) | Analysis 2 |
| Breeding phenology + environmental covariates | Regional data^2^ | Peregrine falcon | Year + Snow + Spring temperature + NDVI | Site/ Breeding territory | Gaussian | Default in brms package (uninformative) | Analysis 3 |
| Breeding phenology + environmental covariates | Regional data^3^ | Gyrfalcon | Year + Snow + Spring temperature + NDVI | Site/ Breeding territory | Gaussian | Default in brms package (uninformative) | Analysis 3 |
| Productivity + environmental covariates | Regional data^2^ | Peregrine falcon | Year + Average yearly hatch date + Snow + Summer temperature + Summer precipitation + NDVI | NA^6^ | Gaussian | Default in brms package (uninformative) | Analysis 4 |
| Productivity + environmental covariates | Regional data^3^ | Gyrfalcon | Year + Average yearly hatch date + Snow + Summer temperature + Summer precipitation + NDVI | NA^6^ | Gaussian | Default in brms package (uninformative) | Analysis 4 |

**Table S2.** Model descriptions used in each analysis within the main text for both gyrfalcon and peregrine falcon hatch date and productivity data.

^a^ Analysis # corresponds to the analyses described in the main text (Methods), allowing direct linkage between model specifications, figures, and results

^1^ Data included both species and all monitoring sites that were grouped by Arctic region (Sub Arctic, Low Arctic, High Arctic)

^2^ Data included only peregrine falcons and all monitoring sites that were grouped by Arctic region (Sub Arctic, Low Arctic, High Arctic)

^3^ Data included only gyrfalcons and all monitoring sites that were grouped by Arctic region (Sub Arctic, Low Arctic, High Arctic)

^4^ Data included only peregrine falcons, and each monitoring site had its own model

^5^ Data included only gyrfalcons, and each monitoring site had its own model

^6^ Random effect of Site was fit for only pooled data

**Table S3.** Univariate results for sources of variation in hatch dates among Arctic regions for peregrine falcons and gyrfalcons. The study period covers 1967-2022 for peregrine falcons and 1973-2022 for gyrfalcons. We fit nestling hatch dates as the response variable, with fixed effects of Arctic region, species, an interaction between Arctic region and species, and random effects of site and year. We report beta estimates (β) as posterior medians, 95% credible intervals, and probability of direction (PD). Effects where credible intervals did not overlap zero were interpreted as having the strongest directional support. Effects where credible intervals overlapped zero but PD was ≥89% were interpreted as having moderate directional support.

| **Nestling hatch date** | | |
| --- | --- | --- |
| **Fixed Effects** | **β**  **(95% CI)** | **PD**  **(%)** |
| Intercept^1^ | 141.5 (138.2, 144.5) | 100 |
| Region |  | |
| Low Arctic | 8.1 (4.0, 12.2) | 100 |
| High Arctic | 22.3 (16.3, 28.4) | 100 |
| Species |  | |
| Peregrine falcon | 33.6 (32.7, 34.7) | 100 |
| Region:Species |  | |
| Low Arctic: Peregrine falcon | 5.0 (3.4, 6.5) | 100 |
| High Arctic: Peregrine falcon | 0.0 (-2.5, 2.7) | 50.37 |
| **Random Effects** | **σ (95% CI)** | |
| Year | 3.8 (1.9, 5.8) | |
| Site ID | 14.9 (5.8, 27.1) | |
| Residual | 50.4 (48.8, 52.1) | |

^1^ Intercept represents Gyrfalcons in the Sub Arctic

**Table S4.** Univariate results for the effect of year on each environmental covariates among Arctic regions. The study period covers 1981-2022. We fit environmental covariates as the response variable and a fixed effects of year. We scaled and centered all environmental covariates and year prior to analysis and report beta estimates (β) as posterior medians, 95% credible intervals, and probability of direction (PD). Effects where credible intervals did not overlap zero were interpreted as having the strongest directional support. Effects where credible intervals overlapped zero but PD was ≥89% were interpreted as having moderate directional support.

|  | | **Spring temperature** | | **Snow** | | **NDVI** | | **Summer temperature** | | **Summer precipitation** | |
| --- | --- | --- | --- | --- | --- | --- | --- | --- | --- | --- | --- |
| **Region** | **Fixed Effects** | **β**  **(95% CI)** | **PD**  **(%)** | **β**  **(95% CI)** | **PD**  **(%)** | **β**  **(95% CI)** | **PD**  **(%)** | **β**  **(95% CI)** | **PD**  **(%)** | **β**  **(95% CI)** | **PD**  **(%)** |
| Sub Arctic | Intercept | 0.1  (-0.6, 0.8) | 58.2 | -0.1  (-1.0, 0.7) | 60.9 | 0.2  (-0.7, 1.0) | 69.5 | 0.2  (-0.9, 1.3) | 68.1 | -0.1  (-0.8, 0.6) | 60.4 |
|  | Year | **0.3**  **(0.2, 0.4)** | 100 | **-0.1**  **(-0.2, 0.0)** | 99.8 | **0.1**  **(0.0, 0.1)** | 98.0 | **0.1**  **(0.0, 0.1)** | 99.1 | **0.1**  **(0.0, 0.2)** | 90.6 |
| Low Arctic | Intercept | 0.1  (-0.4, 0.8) | 71.0 | -0.1  (-0.8, 0.5) | 70.0 | 0.1  (-0.4, 0.8) | 73.3 | 0.2  (-0.8, 1.1) | 72.5 | -0.2  (-1.2, 0.8) | 72.3 |
|  | Year | **0.3**  **(0.1, 0.4)** | 100 | **-0.1**  **(-0.2, 0.0)** | 95.1 | 0.0  (-0.1, 0.1) | 51.1 | **0.2**  **(0.1, 0.4)** | 99.8 | **-0.2**  **(-0.4, -0.1)** | 99.5 |
| Pooled | Intercept | -0.1 (-0.5, 0.4) | 63.9 | 0.0 (-0.4, 0.4) | 52.6 | 0.0 (-0.4, 0.4) | 53.4 | 0.0 (-0.8, 0.7) | 52.4 | -0.2 (-0.7, 0.3) | 83.8 |
|  | Year | **0.2 (0.1, 0.2)** | 100 | **-0.1 (-0.1, 0.0)** | 99.8 | 0.0 (0.0, 0.1) | 75.6 | **0.1 (0.0, 0.1)** | 100 | -0.1 (-0.1, 0.0) | 81.8 |

| **Study Site** | **Site number** | | | **Year** | **Snow** | **Temperature** | **NDVI** |
| --- | --- | --- | --- | --- | --- | --- | --- |
|  |  |  |  | **VIF (95% CI)** | **VIF (95% CI)** | **VIF (95% CI)** | **VIF (95% CI)** |
| **Peregrine** | | | |  |  |  |  |
| South Greenland | | | 2 | 1.12 [1.04, 1.38] | 3.37 [2.80, 4.11] | 3.73 [3.09, 4.56] | 1.22 [1.10, 1.46] |
| Central West Greenland | | | 3 | 1.24 [1.16, 1.35] | 3.00 [2.72, 3.33] | 3.50 [3.16, 3.89] | 1.64 [1.51, 1.79] |
| North Greenland | | | 4 | 2.94 [2.27, 3.96] | 1.18 [1.05, 1.65] | 1.05 [1.00, 2.99] | 3.19 [2.45, 4.32] |
| Mackenzie River (Ca) | | | 5 | 3.68 [3.33, 4.08] | 8.27 [7.40, 9.24] | 4.72 [4.25, 5.26] | 1.40 [1.31, 1.53] |
| North Slope Yukon (Ca) | | | 6 | 1.35 [1.33, 1.38] | 4.27 [4.16, 4.38] | 6.76 [6.57, 6.95] | 2.20 [2.15, 2.25] |
| Yukon/Peel Rivers (Ca) | | | 7 | 1.26 [1.20, 1.35] | 3.42 [3.15, 3.73] | 3.63 [3.33, 3.96] | 1.28 [1.21, 1.37] |
| Upper Yukon (US) | | | 9 | 1.49 [1.39, 1.61] | 1.60 [1.49, 1.73] | 1.89 [1.74, 2.06] | 1.30 [1.23, 1.41] |
| Colville River (US) | | | 10 | 1.03 [1.00, 1.28] | 2.90 [2.61, 3.23] | 2.87 [2.59, 3.20] | 1.03 [1.00, 1.31] |
| Schuchya River (Ru) | | | 14 | 1.13 [1.03, 1.52] | 3.98 [3.13, 5.15] | 3.28 [2.61, 4.22] | 1.47 [1.26, 1.84] |
| Lapland (Fi) | | | 18 | 1.04 [1.01, 1.12] | 1.55 [1.47, 1.64] | 2.29 [2.15, 2.45] | 1.64 [1.55, 1.75] |
| Norrbotten (Se) | | | 19 | 1.01 [1.00, 2.45] | 2.50 [2.17, 2.92] | 2.58 [2.24, 3.02] | 1.46 [1.31, 1.68] |
| **Gyrfalcon** | | | |  |  |  |  |
| Iceland | | 1 | | 1.80 [1.66, 1.97] | 1.75 [1.61, 1.91] | 2.08 [1.91, 2.29] | 2.09 [1.91, 2.29] |
| Central West Greenland | | 3 | | 1.83 [1.62, 2.12] | 6.90 [5.82, 8.22] | 3.16 [2.72, 3.71] | 4.44 [3.78, 5.25] |
| North Greenland | | 4 | | -1.83 [1.54, 2.27] | 1.12 [1.03, 1.48] | 1.03 [1.00, 4.29] | 1.95 [1.64, 2.43] |
| North Slope Yukon (Ca) | | 6 | | 1.26 [1.16, 1.41] | 5.78 [5.02, 6.69] | 1.41 [1.29, 1.58] | 5.74 [4.98, 6.64] |
| Peel Yukon Rivers (Ca) | | 7 | | 1.35 [1.19, 1.62] | 2.33 [1.95, 2.84] | 2.33 [1.95, 2.85] | 1.17 [1.07, 1.44] |
| South Yukon (Ca) | | 8 | | 1.39 [1.25, 1.60] | 1.63 [1.45, 1.88] | 1.67 [1.49, 1.93] | 1.37 [1.24, 1.58] |
| Seward Peninsula (US) | | 11 | | 2.59 [2.40, 2.81] | 2.50 [2.32, 2.71] | 5.83 [5.33, 6.38] | 4.87 [4.46, 5.32] |
| Schuchya River (Ru) | | 14 | | 1.12 [1.04, 1.41] | 2.28 [1.92, 2.78] | 1.16 [1.06, 1.42] | 2.42 [2.03, 2.95] |
| Lapland (Fi) | | 18 | | 1.37 [1.23, 1.59] | 2.96 [2.53, 3.52] | 4.15 [3.50, 4.97] | 1.82 [1.59, 2.13] |

**Table S5.** Variance inflation factor (VIF) results for environmental covariates included in models of hatch date across all long-term monitoring sites for peregrines (*n* =11) and gyrfalcons (*n* = 9) from 1981–2022. This table corresponds to the model results presented in Table 1 of the main text.

**Table S6.** Variance inflation factor (VIF) results for year and environmental covariates included in univariate models of hatch date across Arctic regions for peregrines and gyrfalcons from 1981–2022.

| **Species** | **Region** | **Year** | **Snow** | **Temperature** | **NDVI** |
| --- | --- | --- | --- | --- | --- |
|  |  | **VIF (95% CI)** | **VIF (95% CI)** | **VIF (95% CI)** | **VIF (95% CI)** |
| **Peregrine Falcon** | Sub Arctic | 1.19 (1.15, 1.23) | 2.06 (1.98, 2.14) | 2.24 (2.15, 2.34) | 1.06 (1.04, 1.10) |
|  | Low Arctic | 1.17 (1.12, 1.23) | 2.67 (2.51, 2.84) | 2.68 (2.52, 2.85) | 1.18 (1.13, 1.23) |
|  | Pooled | 1.06 (1.04, 1.09) | 2.16 (2.09, 2.24) | 2.25 (2.18, 2.33) | 1.01 (1.00, 1.19) |
| **Gyrfalcon** | Sub Arctic | 1.59 (1.50, 1.70) | 1.73 (1.62, 1.85) | 2.03 (1.90, 2.18) | 1.75 (1.65, 1.88) |
|  | Low Arctic | 1.29 (1.23, 1.36) | 2.10 (1.98, 2.23) | 2.08 (1.96, 2.21) | 2.10 (1.98, 2.23) |
|  | Pooled | 1.22 (1.18, 1.27) | 1.56 (1.50, 1.63) | 1.63 (1.57, 1.71) | 1.58 (1.52, 1.65) |

**Table S7.** Variance inflation factor (VIF) results for environmental covariates included in univariate models of productivity across Arctic regions for peregrines (1981–2018) and gyrfalcons (1981–2017).

| **Species** | **Region** | **Year** | **Average hatch date** | **Snow** | **Temperature** | **Precipitation** | **NDVI** |
| --- | --- | --- | --- | --- | --- | --- | --- |
|  |  | **VIF (95% CI)** | **VIF (95% CI)** | **VIF (95% CI)** | **VIF (95% CI)** | **VIF (95% CI)** | **VIF (95% CI)** |
| **Peregrine Falcon** | Sub Arctic | 1.37 [1.16, 1.85] | 1.53 [1.26, 2.06] | 1.87 [1.50, 2.53] | 2.72 [2.08, 3.73] | 1.29 [1.11, 1.77] | 2.75 [2.10, 3.77] |
|  | Low Arctic | 1.29 [1.10, 1.85] | 1.85 [1.45, 2.60] | 3.61 [2.63, 5.20] | 1.82 [1.43, 2.56] | 2.46 [1.85, 3.49] | 1.49 [1.22, 2.09] |
|  | Pooled | 1.12 [1.03, 1.48] | 1.42 [1.23, 1.76] | 1.71 [1.45, 2.13] | 1.16 [1.05, 1.49] | 1.19 [1.07, 1.51] | 1.32 [1.16, 1.64] |
| **Gyrfalcon** | Sub Arctic | 1.40 [1.16, 2.00] | 1.67 [1.33, 2.36] | 2.52 [1.88, 3.61] | 2.99 [2.20, 4.32] | 1.19 [1.04, 1.84] | 1.88 [1.47, 2.67] |
|  | Low Arctic | 2.32 [1.69, 3.54] | 1.15 [1.02, 2.20] | 4.99 [3.34, 7.81] | 4.94 [3.30, 7.73] | 1.43 [1.16, 2.21] | 2.64 [1.88, 4.05] |
|  | Pooled | 1.19 [1.06, 1.60] | 1.98 [1.61, 2.58] | 2.21 [1.77, 2.89] | 1.10 [1.01, 1.68] | 1.11 [1.02, 1.63] | 1.51 [1.28, 1.96] |

**Figure S1**. Changes in environmental parameters across years 1982-2022 at falcon monitoring sites in different zones of the Arctic. Each dot contained within each plot represents environmental data from a specific monitoring site and year.
